# Metagenome-Assembled Genomes of Novel Taxa from an Acid Mine Drainage Environment

**DOI:** 10.1128/AEM.00772-21

**Published:** 2021-08-11

**Authors:** Christen L. Grettenberger, Trinity L. Hamilton

**Affiliations:** a Department of Earth and Planetary Sciences, University of California Davis, Davis, California, USA; b Department of Plant and Microbial Biology, University of Minnesota, St. Paul, Minnesota, USA; c The Biotechnology Institute, University of Minnesota, St. Paul, Minnesota, USA; Kyoto University

**Keywords:** AMD, metagenome-assembled genome, biogeochemical cycling, bioremediation, MAG, acid mine drainage, iron, metagenome

## Abstract

Acid mine drainage (AMD) is a global problem in which iron sulfide minerals oxidize and generate acidic, metal-rich water. Bioremediation relies on understanding how microbial communities inhabiting an AMD site contribute to biogeochemical cycling. A number of studies have reported community composition in AMD sites from 16S rRNA gene amplicons, but it remains difficult to link taxa to function, especially in the absence of closely related cultured species or those with published genomes. Unfortunately, there is a paucity of genomes and cultured taxa from AMD environments. Here, we report 29 novel metagenome-assembled genomes from Cabin Branch, an AMD site in the Daniel Boone National Forest, Kentucky, USA. The genomes span 11 bacterial phyla and one archaeal phylum and include taxa that contribute to carbon, nitrogen, sulfur, and iron cycling. These data reveal overlooked taxa that contribute to carbon fixation in AMD sites as well as uncharacterized Fe(II)-oxidizing bacteria. These data provide additional context for 16S rRNA gene studies, add to our understanding of the taxa involved in biogeochemical cycling in AMD environments, and can inform bioremediation strategies.

**IMPORTANCE** Bioremediating acid mine drainage requires understanding how microbial communities influence geochemical cycling of iron and sulfur and biologically important elements such as carbon and nitrogen. Research in this area has provided an abundance of 16S rRNA gene amplicon data. However, linking these data to metabolisms is difficult because many AMD taxa are uncultured or lack published genomes. Here, we present metagenome-assembled genomes from 29 novel AMD taxa and detail their metabolic potential. These data provide information on AMD taxa that could be important for bioremediation strategies, including taxa that are involved in cycling iron, sulfur, carbon, and nitrogen.

## INTRODUCTION

Acid mine drainage (AMD) is a global environmental problem. Oxidative processes, both biotic and abiotic, release protons and reduced metals from sulfide minerals, resulting in highly acidic and toxic conditions that degrade environmental quality. Due to the toxicity and environmental impact of AMD, bioremediation strategies have become of interest. Research in AMD environments often seeks to understand the biogeochemical cycling occurring in the environment and aims to inform and improve the bioremediation of these sites (1–4). Biotic oxidation of reduced metal sulfides contributes to the formation of AMD, while sulfate and iron reduction can both decrease the concentration of soluble metals and increase pH (5–11). However, the metabolic potential of many taxa in AMD environments remains uncharacterized because these taxa are not closely related to cultured taxa or those with published genomes.

AMD environments are characterized by redox gradients including contrasting concentration of oxygen and reduced metals. They can also vary in heavy metal content and pH. However, 16S rRNA gene surveys reveal that many of the same species inhabit AMD sites across the globe. For example, *Ferrovum* spp. are found in the Appalachian Coal Belt (12–14), the Iberian Pyrite Belt (15, 16), Wales (17, 18), and southeast and southwest China (19, 20). *Archaea* within the order *Thermoplasmatales* are commonly found in AMD sites worldwide, especially those with low pH (9, 21–23). In many instances, 16S rRNA sequences isolated from AMD are only distantly related to cultured *Thermoplasmatales*. Taxa in the *Thermoplasmatales* perform diverse metabolic functions, including Fe(II) oxidation (24), obligate heterotrophy (25, 26), and sulfur respiration (27). Therefore, it is difficult to infer their metabolic potential (22). Similarly, taxa within newly discovered phyla such as the *Elusimicrobiota* (formerly Termite Group 1) and *Eremiobacteriota* (formerly the WPS-2) inhabit AMD sites (28, 29), but these groups have few if any cultured taxa. Given the widespread distribution of these lineages, these taxa may play an important role in biogeochemical cycling in AMD environments, but without closely related cultured relatives or well-annotated genomes, it is not possible to elucidate their role or potential use in bioremediation strategies. Even in well-studied AMD groups such as the *Gammaproteobacteria*, multiple closely related taxa may occur in AMD sites but may play different roles from their close relatives. For example, multiple *Ferrovum* taxa differ in their ability to fix nitrogen (30–33). This intragenus metabolic diversity complicates our ability to understand biogeochemical cycling in AMD environments.

Obtaining a species in pure culture has long been considered the gold standard for determining the biogeochemical role that a taxon may play in the environment. However, characterized isolates from AMD environments are rare. Culturing taxa is inherently time-consuming, especially those that require micro-oxic conditions, and can be difficult because species often require cooccurring taxa. For example, in culture, *Ferrovum* spp. cooccur with heterotrophic organisms that remove pyruvic acid and other organic material (34). Metagenomic sequencing has proven to be a valuable tool for guiding isolation of common AMD microbes through the recovery of near-complete genomes. Tyson et al. used a metagenome-directed approach to isolate a Leptospirillum ferrooxidans sp. capable of nitrogen fixation (35). Metagenomic approaches also provide valuable information about community structure and diversity. Thus, ’omics-based approaches can complement pure culture studies, provide valuable insight to biogeochemical cycling in AMD environments, and inform bioremediation strategies in the absence of fully characterized isolates.

Here, we present 29 novel, high-quality, metagenome-assembled genomes (MAGs) from Cabin Branch, an acid mine drainage site in the Daniel Boone National Forest, Kentucky, USA, which further elucidate potential biogeochemical cycling in AMD ecosystems. Several of the MAGs encode Cyc2 like cytochrome *c* involved in Fe(II) oxidation (36–38) and thus represent potential previously uncharacterized Fe(II)-oxidizing bacteria. For example, MAGs within the recently discovered phyla *Elusimicrobia* and *Eremiobacterota* encode Cyc2. These taxa have not previously been recognized as Fe(II) oxidizers, but the recovery of Cyc2 in these MAGs further expands our knowledge of the taxonomic diversity of Fe(II) oxidation. Furthermore, MAGs encoding Cyc2 were recovered across sample sites that range in dissolved oxygen and Fe(II) concentration from 77 to 401 μmol/liter and 11 to 882 μmol/liter, respectively, and are present at relative abundances that suggest key roles in community function. Collectively, the data highlight the metabolic potential of a number of microbes commonly recovered in 16S rRNA-based studies of AMD. These genomes will provide additional context for gene amplicon studies in AMD environments, aid in culturing these taxa in the future, and could inform AMD bioremediation strategies.

## RESULTS

We sequenced metagenomes from three locations in the outflow channel of Cabin Branch, an acid mine drainage site in the Daniel Boone National Forest in Kentucky. Between 74 and 101 million reads were generated per sample. Of these, between 78% and 81% retained both forward and reverse reads after trimming and quality control. These were assembled into 50,449 to 118,786 contigs with median contig lengths of 1,979 to 3,853 bp. Between 53.9% and 56.0% of reads were successfully mapped back to the assembled contigs. The emergence sample resulted in 38 bins, the outflow, 32, Rose Pool, 66, and the combined assembly, 120. Genome assembly, mapping, and binning statistics are available in File S4 in the supplemental material.

**FIG 1:**
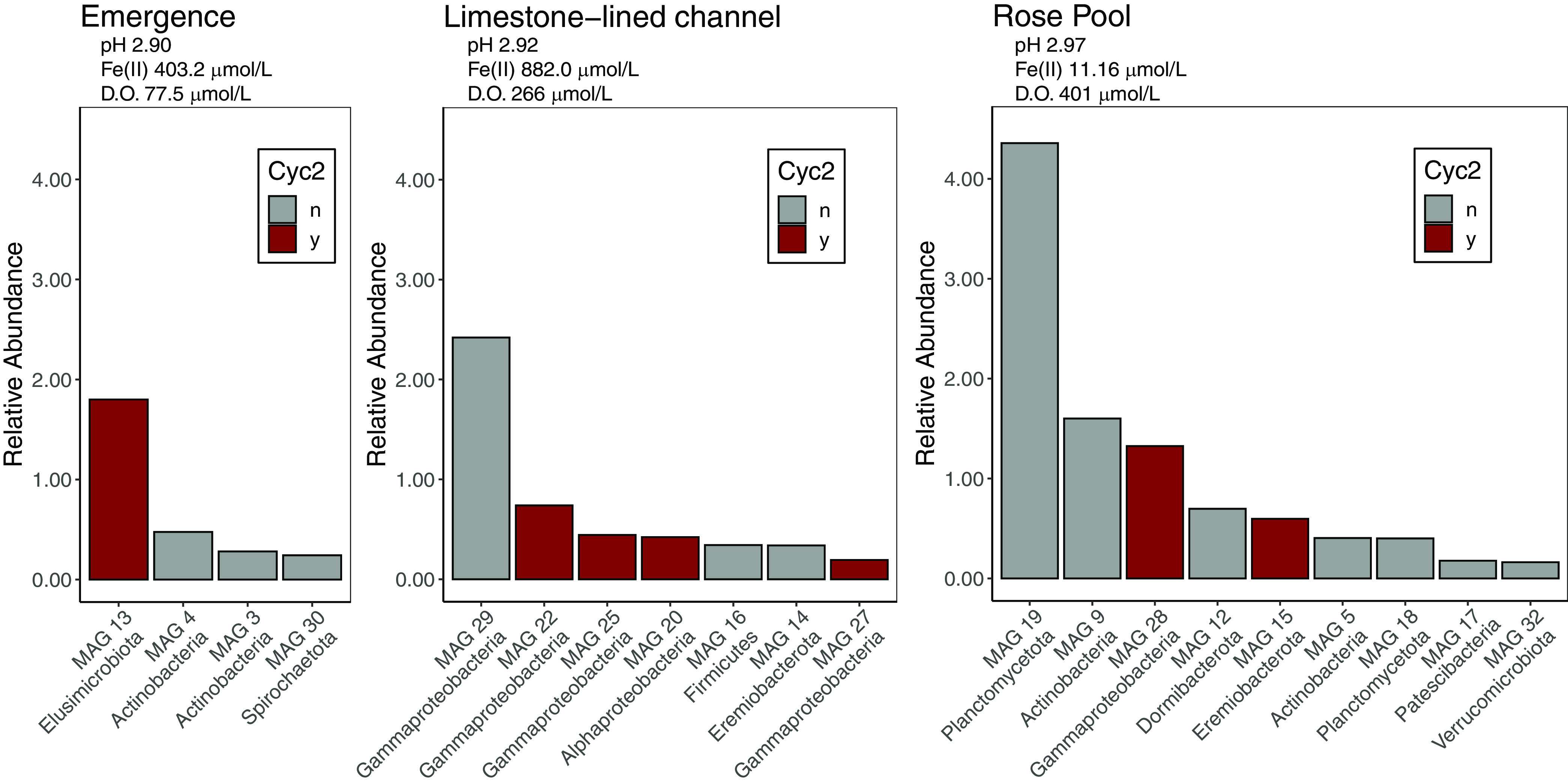
Rank abundance curve of the relative abundance of MAGs recovered from the emergence, the limestone-lined channel, and Rose Pool. pH, Fe(II), and D.O. were measured at the time of sample collection and are reported in reference 33. Red bars indicate MAGs that encode Cyc2. D.O., dissolved oxygen.

**FIG 2:**
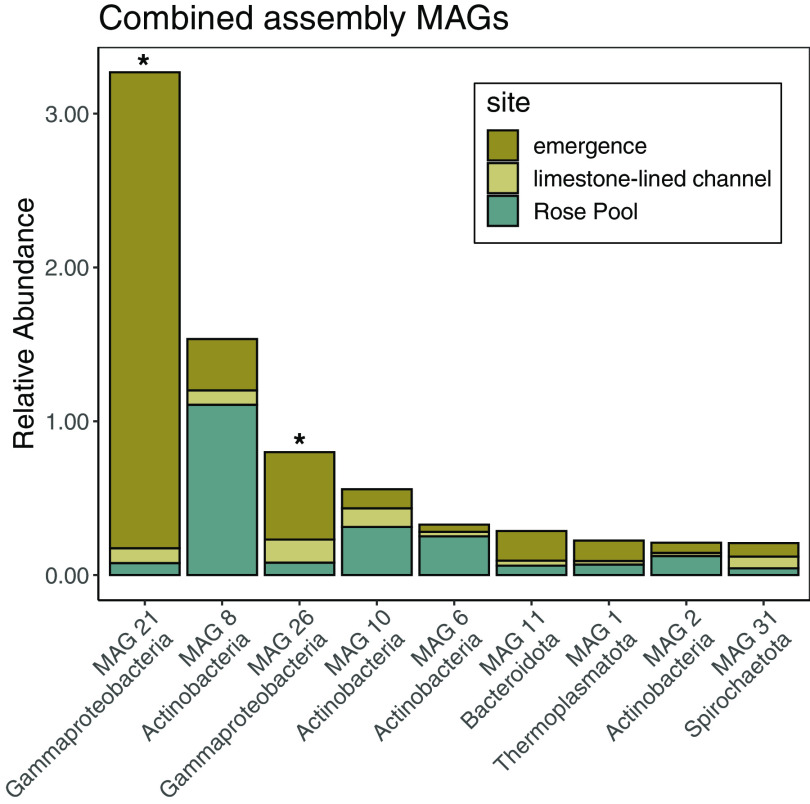
Rank abundance curve of the relative abundance of MAGs recovered from the coassembled data in each site—the emergence, the limestone-lined channel, and Rose Pool. Asterisks denote MAGs that encode Cyc2.

We recovered 256 bins from the metagenomes—38 from the emergence, 32 from the limestone-lined channel, 66 from Rose Pool, and 120 from the coassembly. Of these, 56 were >70% complete with <3% contamination. These bins belonged to 32 unique taxa (Table 1). Here, we present 29 novel, high-quality, metagenome-assembled genomes (MAGs), 4 from the emergence, 7 from the limestone-lined channel, 9 from Rose Pool, and 9 from the coassembled data. The MAGs ranged in relative abundance from ∼4.3% to ∼0.17% (Fig. 1 and 2). The *Ferrovum* MAGs (MAG 23 and MAG24) were described in reference 33, and MAG 7 is closely related to a previously described genome (39, 40).

**TABLE 1:** Summary of the MAGs presented here^*a*^^,^^*b*^

Phylum	MAG	Accession no.	Taxonomy	Completeness (%)	Contamination (%)	Strain heterogeneity	Size (Mbp)	No. of contigs	GC content (%)	No. of protein coding sequences	Carbon fixation	Sulfur cycling	Nitrogen fixation	Fe(II) oxidation
*Thermoplasmatota*	MAG 1	SAMN14771053	*Archaea*; *Thermoplasmatota*; *Thermoplasmata*; UBA184; UBA184	84.36	1.6	0	1.418903	217	0.67	1,457	No	No	No	No
*Actinobacteria*	MAG 2	SAMN14771054	*Bacteria*; *Actinobacteriota*; *Acidimicrobiia*; *Acidimicrobiales*	84.62	2.14	0	2.526595	340	0.45	2,362	No	No	No	No
	MAG 3	SAMN14771055	*Bacteria*; *Actinobacteriota*; *Acidimicrobiia*; *Acidimicrobiales*; *Acidimicrobiaceae*	90.88	0.85	0	2.378105	192	0.52928	2,137	Yes	No	No	No
	MAG 4	SAMN14771056	*Bacteria*; *Actinobacteriota*; *Acidimicrobiia*; *Acidimicrobiales*; *Acidimicrobiaceae*	99.15	1.28	0	2.817353	95	0.55046	2,584	Yes	No	No	No
	MAG 5	SAMN14771057	*Bacteria*; *Actinobacteriota*; *Acidimicrobiia*; *Acidimicrobiales*; *Acidimicrobiaceae*	85.13	1.36	33.33	2.138544	268	0.53278	1,873	Yes	No	No	No
	MAG 6	SAMN14771058	*Bacteria*; *Actinobacteriota*; *Acidimicrobiia*; *Acidimicrobiales*; *Acidimicrobiaceae*	94.02	2.14	0	2.323875	280	0.50685	2,129	Yes	No	No	No
	*MAG 7*		*Bacteria; Actinobacteriota; Acidimicrobiia; Acidimicrobiales; Acidimicrobiaceae; Acidithrix; Acidithrix ferrooxidans*	*70.36*	*2.14*	*33.33*	*2.417212*	*336*	*0.47572*	*2,062*	NA	NA	NA	NA
	MAG 8	SAMN14771059	*Bacteria*; *Actinobacteriota*; *Acidimicrobiia*; *Acidimicrobiales*; RAAP-2; RAAP-2	96.3	2.99	0	2.796923	246	0.59901	2,749	No	No	No	No
	MAG 9	SAMN14771060	*Bacteria*; *Actinobacteriota*; *Acidimicrobiia*; *Acidimicrobiales*; RAAP-2; RAAP-2	93.08	2.23	0	2.2056	175	0.6155	2,197	Yes	No	No	No
	MAG 10	SAMN14771061	Bacteria; Actinobacteriota; Acidimicrobiia; Acidimicrobiales; RAAP-2; RAAP-2;	94.87	2.14	0	1.873312	163	0.63812	1,856	No	No	No	No
*Bacteroidota*	MAG 11	SAMN14771062	*Bacteria*; *Bacteroidota*; *Bacteroidia*; AKYH767; Palsa-948	84.22	2	40	2.420414	335	0.46586	2,125	No	No	No	No
*Dormibacterota*	MAG 12	SAMN14771063	*Bacteria*; *Dormibacterota*; *Dormibacteria*; UBA8260; Bog-877	95.37	0.93	0	2.296128	226	0.68404	2,207	No	No	No	No
*Elusimicrobiota*	MAG 13	SAMN14771064	*Bacteria*; *Elusimicrobiota*; *Elusimicrobia*; UBA1565; UBA9628; GWA2-66-18	86.06	1.5	0	3.360875	299	0.6663	3,200	No	No	Yes	Yes
*Eremiobacterota*	MAG 14	SAMN14771065	*Bacteria*; *Eremiobacterota*; *Eremiobacteria*; UBP12; UBA5184	71.43	2.78	100	1.980657	269	0.62541	1,976	No	No	No	No
	MAG 15	SAMN14771066	*Bacteria*; *Eremiobacterota*; *Eremiobacteria*; UBP12; UBA5184	79.18	1.85	50	2.083017	215	0.62452	2,102	No	No	No	Yes
*Firmicutes*	MAG 16	SAMN14771067	*Bacteria*; *Firmicutes_K*; *Alicyclobacillia*; *Alicyclobacillales*; *Acidibacillaceae*;	92.65	1.57	50	2.540112	156	0.45602	2,410	No	No	No	No
	MAG 17	SAMN14771068	*Bacteria*; *Patescibacteria*; *Paceibacteria*; UBA9983_A; UBA2163; C7867-001	71.84	0	0	2.138544	268	0.53278	1,873	No	No	No	No
*Planctomycetota*	MAG 18	SAMN14771069	*Bacteria*; *Planctomycetota*; *Phycisphaerae*	73.45	0	0	2.799192	419	0.56703	2,424	No	No	No	No
	MAG 19	SAMN14771070	*Bacteria*; *Planctomycetota*; *Phycisphaerae*; UBA1161	96.32	0	0	3.578576	255	0.56229	2,916	No	No	No	No
*Proteobacteria*	MAG 20	SAMN14771071	*Bacteria*; *Proteobacteria*; *Alphaproteobacteria*; *Acetobacterales*; *Acetobacteraceae*; *Acidocella*	92.45	2.78	57.14	2.678786	222	0.63102	2,577	Yes	No	Yes	Yes
	MAG 21	SAMN14771072	*Bacteria*; *Proteobacteria*; *Gammaproteobacteria*; *Burkholderiales*	91.83	2.01	20	2.555995	242	0.57887	2,573	Yes	Yes	Yes	Yes
	MAG 22	SAMN14771073	*Bacteria*; *Proteobacteria*; *Gammaproteobacteria*; *Burkholderiales*	99.14	0	0	3.695939	108	0.64413	3,442	Yes	Yes	No	Yes
	*MAG 23*		*Bacteria; Proteobacteria; Gammaproteobacteria; Burkholderiales; Ferrovaceae*	*95.76*	*0.5*	*0*	*2.374093*	*102*	*0.55802*	*2,182*	NA	NA	NA	NA
	*MAG 24*		*Bacteria; Proteobacteria; Gammaproteobacteria; Burkholderiales; Ferrovaceae; Ferrovum*	*86.7*	*0.63*	*100*	*1.845635*	*96*	*0.5385*	*1,727*	NA	NA	NA	NA
	MAG 25	SAMN14771074	*Bacteria*; *Proteobacteria*; *Gammaproteobacteria*; *Burkholderiales*; *Gallionellaceae*; *Gallionella*	96.67	1.43	0	2.478987	157	0.5653	2,425	Yes	No	No	Yes
	MAG 26	SAMN14771075	*Bacteria*; *Proteobacteria*; *Gammaproteobacteria*; *Burkholderiales*; *Gallionellaceae*; *Gallionella*	94.44	2.41	83.33	2.296128	226	0.68404	2,207	Yes	No	No	Yes
	MAG 27	SAMN14771076	*Bacteria*; *Proteobacteria*; *Gammaproteobacteria*; *Pseudomonadales*	91.16	1.24	14.29	2.572997	319	0.51007	2,364	No	No	Yes	Yes
	MAG 28	SAMN14771077	*Bacteria*; *Proteobacteria*; *Gammaproteobacteria*; *Steroidobacterales*; *Steroidobacteraceae*;	85.78	2.5	18.18	2.564032	320	0.65702	2,382	Yes	No	No	Yes
	MAG 29	SAMN14771078	*Bacteria*; *Proteobacteria*; *Gammaproteobacteria*; UBA1113; UBA1113	93.6	1.36	33.33	2.699483	337	0.39968	2,364	No	No	No	No
*Spirochaetota*	MAG 30	SAMN14771079	*Bacteria*; *Spirochaetota*; *Spirochaetia*; *Spirochaetales*	95.4	0.4	0	2.61822	142	0.50392	2,361	No	No	No	No
	MAG 31	SAMN14771080	*Bacteria*; *Spirochaetota*; *Spirochaetia*; *Spirochaetales*	77.93	1.6	50	1.946496	327	0.53893	1,830	No	No	No	No
*Verrucomicrobiota*	MAG 32	SAMN14771081	*Bacteria*; *Verrucomicrobiota_A*; *Chlamydiia*; *Parachlamydiales*; Ga0074140	87.1	1.35	50	1.881114	247	0.39274	1,615	No	No	No	No

a Taxa in italic text were not analyzed in this work because they were either closely related to cultured taxa (e.g., MAG 7) or presented previously (MAGs 24 and 24 [33]).

b NA, not analyzed.

Below, we examine functions that are most relevant to AMD ecosystems, including aerobic respiration, carbon fixation, nitrogen cycling, and biogeochemical cycling of sulfur and iron in each MAG by phylogenetic group. For sulfur cycling, we focus on dissimilatory sulfate reduction and sulfur oxidation by examining the presence or absence of *dsr* and *sox* genes. The metabolic potential for ferrous iron oxidation was based on the presence of *Cyc2*-like genes that may be involved in this process (36, 37). None of the MAGs contain complete genomes, and a gene that is absent in the MAG may be present in the taxon. Therefore, these data indicate the genes present in, not absent from, a taxon. A summary of these taxa is available in File S5. More complete genome descriptions are available in the supplemental material, and the METABOLIC and DRAM results are provided in File S6 and Data Set S1.

### Bacteria.

**FIG 3:**
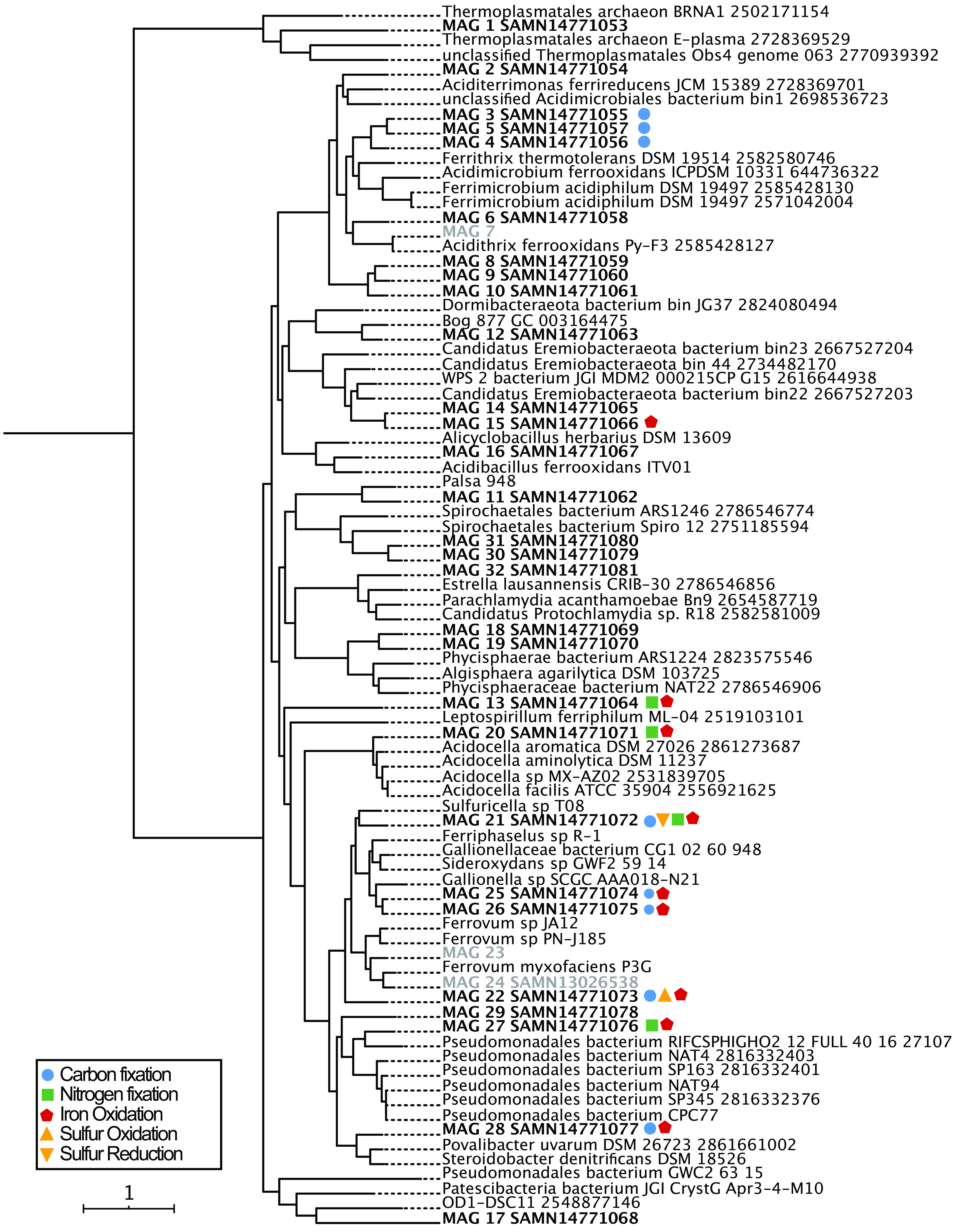
Concatenated single-copy marker gene tree constructed using genes from reference 46. The tree contains 83 taxa. MAGs from Cabin Branch are indicated in bold. Shapes indicate the metabolic potential of the MAGs. Carbon fixation is represented by blue circles. Dissimilatory sulfur oxidation is represented by yellow triangles with points up and reduction, by triangles with points down, nitrogen fixation is represented by green squares, and Fe(II) oxidation is represented by red pentagons. The concatenated single-copy marker gene tree is provided in Newick format in File S1.

**(i) *Actinobacteriota* (*Actinobacteria*).** We retrieved nine actinobacterial MAGs, all of which belong to the order *Acidimicrobiales* (Fig. 3). MAGs 3 to 7 belonged to the *Acidimicrobiaceae*, but MAGs 3 to 6 were unidentified below this level. MAGs 8 to 10 were affiliated with the family and genus RAAP-2 but were unidentified at the species level. Five taxa (MAGs 3 to 6 and 9) encode genes for carbon fixation. *Actinobacteria* MAGs were recovered from the emergence and Rose Pool (Fig. 1) as well as the coassembly (Fig. 2).

*(a) MAG 2.* MAG 2 belonged to the order *Acidimicrobiales* (taxonomy was not resolved below this level) (Fig. 1). The only bin from this taxon was retrieved from the coassembly. It contained 2.53 Mbp in 340 contigs with 2,362 protein-coding genes. The MAG was 84.6% complete with 2.14% contamination, and the GC content was 45% (Table 1). It encodes many of the genes necessary for oxidative phosphorylation with oxygen as a terminal electron acceptor using a *caa*_3_-type cytochrome *c* oxidase. It also contains the genes necessary for conversion of nitrate to nitrite and the reverse reaction (File S6 and Data Set S1).

*(b) MAG 3*. The only bin from MAG 3 was retrieved from the coassembly. It contained 2.38 Mbp in 192 contigs with 2,137 protein-coding genes. It was 90.9% complete with 0.85% contamination. The GC content was 53% (Table 1). The genome encodes homologs of the genes necessary for carbon fixation, including those for form I RuBisCO and for the Wood Ljungdahl pathway. It also encodes many of the genes necessary for oxidative phosphorylation with oxygen as a terminal electron acceptor using *caa*_3_- and *cbb*_3_-type cytochrome *c* oxidases (Data Set S1 and File S6).

*(c) MAG 4*. MAG 4 was retrieved from metagenomes from the emergence, Rose Pool, and the coassembly. The most complete bin from this taxon was retrieved from the emergence. It contained 2.82 Mbp in 95 contigs with 2,584 protein-coding genes. The MAG was 99.2% complete with 1.28% contamination, and the GC content was 55% (Table 1). MAG 4 encodes the genes necessary for oxidative phosphorylation with a *caa*_3_- or *cbb*_3_-type cytochrome *c* oxidase and carbon fixation with form I RuBisCO. It also contains homologs of the genes necessary for mercury reduction (File S6 and Data Set S1).

*(d) MAG 5*. The only bin from MAG 5 was retrieved from Rose Pool. It contained 2.14 Mbp in 268 contigs with 1,873 protein-coding genes. The MAG was 85.1% complete with 1.36% contamination, and the GC content was 53% (Table 1). MAG 5 encodes homologs of the genes necessary for oxidative phosphorylation using either a *caa*_3_- or a *bd*-type cytochrome *c* oxidation as a terminal electron acceptor. It also encodes many of those necessary for carbon fixation using form I RuBisCO and via the 3 hydroxypropionate cycle (File S6 and Data Set S1).

*(e) MAG 6*. Bins of MAG 6 were retrieved from Rose Pool and the coassembly. The most complete bin from this taxon was retrieved from the coassembly. It contained 2.32 Mbp in 280 contigs with 2,129 protein-coding genes. The MAG was 94% complete with 2.14% contamination, and the GC content was 51% (Table 1). MAG 6 encodes homologs of many of the genes necessary for oxidative phosphorylation using a *caa*_3_- or *cbb*_3_-type cytochrome *c* oxidase as a terminal electron acceptor (File S6 and Data Set S1).

*(f) MAG 7*. MAG 7 was closely related to Acidithrix ferrooxidans (96.96% average nucleotide identity [ANI]). This taxon was described elsewhere (1, 2), and therefore we do not discuss it here.

*(g) MAG 8*. Bins of MAG 8 were retrieved from Rose Pool and the coassembly. The most complete bin from MAG 8 was retrieved from the coassembly. It contained 2.80 Mbp in 246 contigs with 2,749 protein-coding genes. The MAG was 96.3% complete with 2.99% contamination, and the GC content was 60% (Table 1). MAG 8 encodes many of the genes necessary for oxidative phosphorylation using *caa*_3_-, *cbb*_3_-, and *bd*-type cytochrome *c* oxidases (File S6 and Data Set S1).

*(h) MAG 9*. The only bin of MAG 9 was retrieved from Rose Pool. It contained 2.20 Mbp in 175 contigs with 2,197 protein-coding genes. The MAG was 93.1% complete with 2.23% contamination, and the GC content was 62% (Table 1). It contains homologs of some of the genes necessary for oxidative phosphorylation using *cbb*_3_- and *bd* type cytochrome *c* oxidases (File S6 and Data Set S1).

*(i) MAG 10*. The only bin of MAG 10 was retrieved from the coassembly. It contained 1.87 Mbp in 163 contigs with 1,856 protein-coding genes. The MAG was 94.9% complete with 2.14% contamination, and the GC content was 64% (Table 1). MAG 10 encodes homologs for some of the genes necessary for oxidative phosphorylation using *caa*_3_- and *cbb*_3_-type cytochrome *c* oxidases (Data Set S1 and File S6).

### (ii) *Bacteroidota.*

We retrieved a single taxon from the phylum *Bacterioidota* (*Bacteroidetes*; MAG 11). Bins from MAG 11 were retrieved from the emergence and coassembly. It was affiliated with the class *Bacteroidia*, order AKH767, and family Palsa-948 and was unclassified below this level. The most closely related taxon was retrieved from thawing permafrost (41). The best bin from this taxon was retrieved from the coassembly. The most complete bin contained 2.42 Mbp in 335 contigs with 2,125 protein-coding genes. The MAG was 84.2% complete with 2.0% contamination, and the GC content was 47% (Table 1). This taxon encodes some of the genes necessary for oxidative phosphorylation but does not encode any genes for terminal oxidases. It also encodes the genes necessary for arsenate reduction (Data Set S1 and File S6).

### (iii) *Dormibacteraeota.*

We retrieved one taxon from the phylum *Dormibacteraeota* (MAG 12). Bins from MAG 12 were retrieved from Rose Pool and coassembly. This taxon was affiliated with the class *Dormibacteria*, the order UBA8260, and the family Bog-877 and is most closely related to a taxon from thawing permafrost (41). The most complete bin from this taxon was retrieved from Rose Pool. It contained 2.30 Mbp in 226 contigs with 2,207 protein-coding genes. The MAG was 95.4% complete with 0.93% contamination, and the GC content was 68% (Table 1). This taxon encodes some of the genes necessary for oxidative phosphorylation using a *caa*_3_-type cytochrome *c* oxidase as a terminal electron acceptor. It did not encode genes for carbon fixation, N_2_ fixation, denitrification, dissimilatory sulfur cycling, or Fe(II) oxidation (Data Set S1 and File S6).

### (iv) *Elusimicrobiota.*

We retrieved a single taxon, MAG 13, from the phylum *Elusimicrobiota* (formerly Termite Group 1). Bins of MAG 13 were retrieved from the emergence and coassembly. In the emergence, it was present with a relative abundance of ∼1.8%. It was affiliated with the class *Elusimicrobia*, order UBA1565, family UBA9628, and genus GWA2-66-18. This taxon is most closely related to one from an aquifer system (42). The most complete bin from this taxon was retrieved from the emergence. It contained 3.36 Mbp in 299 contigs with 3,200 protein-coding genes. It was 86.1% complete with 1.5% contamination. The GC content was 67% (Table 1). MAG 13 encodes many of the genes necessary for oxidative phosphorylation with *caa*_3_-, *cbb*_3_-, and *bd*-type cytochrome *c* oxidases. It encodes the genes necessary for N_2_ fixation and nitric oxide reduction (Table 1). It also encodes the genes necessary for arsenate reduction (Data Set S1 and File S6) and encodes a Cyc2 like cytochrome that it may use for Fe(II) oxidation (File S2).

### (v) *Eremiobacteriota.*

We retrieved two taxa, MAGs 14 and 15, within the phylum *Eremiobacteriota* (formerly WPS-2). Both taxa were affiliated with the class *Eremiobacteria*, order UPB12, and family UBA5184. Neither bin contained genes for carbon fixation, N_2_ fixation, denitrification, dissimilatory sulfur cycling, or Fe(II) oxidation. Both taxa appear to be capable of heterotrophic metabolism, including aromatics degradation and acetogenesis. MAG 14 was recovered from the limestone-lined channel while MAG 15 was present in Rose Pool (Fig. 1).

*(a) MAG 14*. The only bin from MAG 14 was retrieved from the limestone-lined channel. It contained 1.98 Mbp in 269 contigs with 1,976 protein-coding genes. The MAG was 71.4% complete with 2.78% contamination, and the GC content was 63% (Table 1). MAG 14 encodes many of the genes necessary for oxidative phosphorylation using *caa*_3_- and *bd*-type cytochrome *c* oxidases (Data Set S1 and File S6).

*(b) MAG 15*. The only bin from MAG 15 was retrieved from Rose Pool. It contained 2.08 Mbp in 215 contigs with 2,102 protein-coding genes. The MAG was 79.2% complete with 1.85% contamination, and the GC content was 62% (Table 1). MAG 15 encodes many of the genes necessary for oxidative phosphorylation using a *bd*-type cytochrome *c* oxidase (Data Set S1 and File S6). It also appears to encode a Cyc2-like cytochrome that it may use for Fe(II) oxidation (File S2).

### (vi) *Firmicutes.*

We retrieved a single taxon from the phylum *Firmicutes* (MAG 16) from the limestone-lined channel and the coassembly (Fig. 1). This taxon was affiliated with the class *Alicyclobacillia*, the order *Alicyclobacillales*, and the family *Acidibacillaceae*. This taxon was most closely related to Acidobacillus ferrooxidans, a Fe(II) and sulfide mineral oxidizing species isolated from an AMD environment (Fig. 1) (43). The most complete bin from this taxon was retrieved from the limestone-lined channel. It contained 2.54 Mbp in 156 contigs with 2,410 protein-coding genes. The MAG was 92.7% complete with 1.57% contamination, and the GC content was 46% (Table 1). The genome encodes some of the genes necessary for oxidative phosphorylation using *bo*-, *bd*- and *caa*_3_-type cytochrome *c* oxidases. It also encodes the genes necessary for denitrification (Data Set S1 and File S6). Unlike its closest relative, it does not encode the genes necessary for Fe(II) or sulfide mineral oxidation.

### (vii) *Patescibacteria.*

We retrieved a single taxon, MAG 17, from the phylum *Patescibacteria* (formerly Candidate Phylum Radiation) from Rose Pool (Fig. 1). This taxon was affiliated with the class *Paceibacteria*, the order UBA9983_A, the family UBA2163, and the genus C7867-001. It contained 2.13 Mbp in 268 contigs with 1,873 protein-coding genes. It was 71.84% complete with 0% contamination. The GC content was 53% (Table 1).

**(viii) *Planctomycetota* (*Planctomycetes*).** We recovered two taxa from the phylum *Planctomycetota* (*Planctomycetes*), MAGs 18 and 19. Both taxa were affiliated with the class *Phycisphaerae*. One, MAG 18, was unclassified below this level. The other, MAG 19, was affiliated with the order UBA1161. Both MAG 18 and 19 were recovered from Rose Pool, where MAG 19 was more abundant (4.3% and 0.4%, respectively, Fig. 1).

*(a) MAG 18*. The only bin from this taxon was retrieved from Rose Pool. It contained 2.80 Mbp in 419 contigs with 2,424 protein-coding genes. It was 73.5% complete with 0% contamination. The GC content was 57% (Table 1). MAG 18 encodes some of the genes necessary for oxidative phosphorylation using a *caa*_3_- or *bd-*type cytochrome *c* oxidase (Data Set S1 and File S6).

*(b) MAG 19*. Bins from this taxon were recovered from Rose Pool and the coassembly. The most complete bin from this taxon was retrieved from Rose Pool. It contained 3.58 Mbp in 255 contigs with 2,916 protein-coding genes. The MAG was 96.3% complete with 0% contamination, and the GC content was 56% (Table 1). The MAG encodes some of the genes necessary for oxidative phosphorylation using a *caa*_3_- or *bd-*type cytochrome *c* oxidase (Data Set S1 and File S6).

### (ix) *Proteobacteria.*

We recovered 10 taxa from the *Proteobacteria*. One, MAG 20, is a member of the *Alphaproteobacteria*. Nine are from the gammaproteobacterial order *Burkholderiales*. Two of these were described previously and were not analyzed here (33). Three encode genes for N_2_ fixation (MAGs 20, 21, and 27), six encode genes for carbon fixation, (MAGs 20 to 22, 25, 26, and 28), seven encode genes for Fe(II) oxidation (MAGs 20 to 22 and 25 to 28), and three encode genes for partial sulfate reduction (MAGs 22, 25, and 26). Gammaproteobacterial MAGs were recovered from the limestone-lined channel, Rose Pool, and the coassembly (Fig. 1 and 2). MAG 21 from the coassembly was particularly abundant at the emergence (3.3%), while MAG 29 was abundant in the limestone-lined channel (2.4%).

*(a) MAG 20.* MAG 20 is from the alphaproteobacterial order *Acetobacterales* and is affiliated with the family *Acetobacteraceae* and the genus *Acidocella*. The only bin from this taxon was retrieved from the limestone-lined channel. It contained 2.67 Mbp in 222 contigs with 2,577 protein-coding genes. The MAG was 92.5% complete with 2.78% contamination, and the GC content was 63% (Table 1). MAG 20 encodes the genes necessary for oxidative phosphorylation using *caa*_3_- and *bd-*type cytochrome *c* oxidases. It encodes homologs of genes necessary for N_2_ fixation. It also encodes the genes necessary for mercury reduction (Data Set S1 and File S6). It may also encode a Cyc2-like cytochrome that it may use for Fe(II) oxidation (File S2).

*(b) MAG 21.* MAG 21 is from the gammaproteobacterial order *Burkholderiales* but is unclassified below this level. Bins from this taxon were retrieved from the emergence and the coassembly. The best bin from this taxon was retrieved from the coassembly. It contained 2.55 Mbp in 242 contigs with 2,573 protein-coding genes. The MAG was 91.8% complete with 2.01% contamination, and the GC content was 57% (Table 1). MAG 21 encodes the genes necessary for oxidative phosphorylation using a *bo*- or *bd-*type cytochrome *c* oxidase. This taxon encodes homologs of genes for a form II RuBisCO for carbon fixation and encodes the genes necessary for N_2_ fixation. The genome encoded homologs of *dsrA*, *dsrB*, and *aprA*, indicating that it may be capable of dissimilatory sulfate reduction, at least from APS (adenosine 5′-phosphosulfate) to sulfide. It also encodes the genes necessary for the breakdown of halogenated compounds and arsenate reduction (Data Set S1 and File S6). It may also encode a Cyc2-like cytochrome that it may use for Fe(II) oxidation (File S2).

*(c) MAG 22.* Bins from this taxon were retrieved from the emergence, limestone-lined channel, and coassembly. The best bin from this taxon was retrieved from the limestone-lined channel. It contained 3.70 Mbp in 108 contigs with 3,442 protein-coding genes. The MAG was 99.1% complete with 0% contamination, and the GC content was 64% (Table 1). MAG 22 encodes homologs of the genes necessary for oxidative phosphorylation using *caa*_3_- and *bd-*type cytochrome *c* oxidases. The MAG contains genes necessary for carbon fixation with a form I RuBisCO. It also encodes the genes necessary for the oxidation of thiosulfate to sulfate (*soxB*, *soxY*, and *soxC*) and for mercury reduction (File S5). MAG 22 also appears to encode a Cyc2-like cytochrome that it may use for Fe(II) oxidation (Data Set S1 and File S6).

*(d) MAGs 23 and 24.* These taxa were members of the genus *Ferrovum* and were described elsewhere (33). Therefore, we do not discuss them here.

*(e) MAG 25.* Bins from this taxon were retrieved from the emergence, limestone-lined channel, and coassembly. The best bin from this taxon was retrieved from the limestone-lined channel. It contained 2.48 Mbp in 157 contigs with 2,425 protein-coding genes. The MAG was 96.7% complete with 1.43% contamination, and the GC content was 57% (Table 1). MAG 25 encodes the genes necessary for oxidative phosphorylation but does not encode any terminal oxidases. It also contains the genes necessary for carbon fixation, including type I and II RuBisCOs (Data Set S1 and File S6). It appears to encode a Cyc2 like cytochrome that it may use for Fe(II) oxidation (File S2).

*(f) MAG 26.* Bins from this taxon were retrieved from the emergence and coassembly. The most complete bin from this taxon was retrieved from the coassembly. It contained 2.30 Mbp in 226 contigs with 2,207 protein-coding genes. The MAG was 94.4% complete with 2.41% contamination, and the GC content was 68% (Table 1). It encodes the genes necessary for oxidative phosphorylation using *cbb*_3_- and *bd-*type cytochrome *c* oxidases. It also contains the genes for type I and II RuBisCO for carbon fixation (Data Set S1 and File S6). It appears to encode a Cyc2-like cytochrome that it may use for Fe(II) oxidation (File S2).

*(g) MAG 27.* The only bin from this taxon was retrieved from the limestone-lined channel. It contained 2.57 Mbp in 319 contigs with 2,364 protein-coding genes. The MAG was 91.1% complete with 1.24% contamination, and the GC content was 51% (Table 1). MAG 27 encodes the genes for oxidative phosphorylation using a *cbb*_3_-type cytochrome *c* oxidase. It encodes the genes necessary for N_2_ fixation (Data Set S1 and File S6). It may also encode a Cyc2 like cytochrome that it may use for Fe(II) oxidation (File S2).

*(h) MAG 28.* Bins from this taxon were retrieved from Rose Pool and the coassembly. The best bin from this taxon was retrieved from Rose Pool. It contained 2.56 Mbp in 320 contigs with 2,382 protein-coding genes. The MAG was 85.8% complete with 2.50% contamination, and the GC content was 66% (Table 1). MAG 28 encodes the genes necessary for oxidative phosphorylation using *caa*_3_- and *bo-*type cytochrome *c* oxidases (Data Set S1 and File S6). It may also encode a Cyc2-like cytochrome that it may use for Fe(II) oxidation (File S2).

*(i) MAG 29.* Bins from this taxon were retrieved from the limestone-lined channel and coassembly. The most complete bin from this taxon was retrieved from the limestone-lined channel. It contained 2.70 Mbp in 337 contigs with 2,364 protein-coding genes. The MAG was 93.6% complete with 1.36% contamination, and the GC content was 40% (Table 1). MAG 29 encodes most of the genes necessary for oxidative phosphorylation using *bo*- and *bd-*type cytochrome *c* oxidases (Data Set S1 and File S6). It does not encode the genes necessary for carbon fixation or any other genes of interest.

### (x) *Spirochaetota* (*Spirochaetes*).

We recovered two taxa from the phylum *Spirochaetota* (*Spirochaetes*; MAGs 30 and 31) from Rose Pool (Fig. 1). MAG 30 was recovered from the emergence, and MAG 31 was present in the coassembly with low relative abundance across all sites (Fig. 1 and 2). Both bins were members of the class *Spirochaetia* and the order *Spirochaetales*. One encodes the genes necessary for nitrate reduction to ammonia.

*(a) MAG 30.* Bins from this taxon were retrieved from the emergence, limestone-lined channel, and coassembly. The best bin from this taxon was retrieved from the emergence. It contained 2.62 Mbp in 142 contigs with 2,361 protein-coding genes. The MAG was 95.4% complete with 0.4% contamination, and the GC content was 50% (Table 1). MAG 30 encodes the genes for *bo*- and *bd* type cytochrome *c* oxidases but does not contain other genes associated with oxidative phosphorylation. It encodes the genes necessary for nitrification and denitrification (Data Set S1 and File S6).

*(b) MAG 31.* The only bin from this taxon was retrieved from the coassembly. It contained 1.95 Mbp in 327 contigs with 1,830 protein-coding genes. The MAG was 77.9% complete with 1.6% contamination, and the GC content was 54% (Table 1). MAG 31 encodes the genes necessary for a *caa*_3_-type cytochrome *c* oxidase but does not contain other genes associated with oxidative phosphorylation (Data Set S1 and File S6).

**(xi) *Verrucomicrobiota* (*Verrucomicrobia*).** We recovered a single taxon from the *Verrucomicrobiota* (*Verrucomicrobia*; MAG 32).

Bins from this taxon were retrieved from Rose Pool and the coassembly. The most complete bin from this taxon was retrieved from Rose Pool. It contained 1.88 Mbp in 247 contigs with 1,615 protein-coding genes. The MAG was 87.1% complete with 1.35% contamination, and the GC content was 39% (Table 1). MAG 32 encodes some of the genes necessary for oxidative phosphorylation using *bo*- or *bd-*type cytochrome *c* oxidases (Data Set S1 and File S6).

### Archaea.

We recovered a single MAG classified as *Archaea* (MAG 1). The MAG belonged to the *Thermoplasmata* and was most closely related to *Methanomassiliicoccus* spp. (Fig. 1). The most complete bin contained 1.42 Mbp in 217 contigs with 1,457 protein-coding genes. It was 84.3% complete with 1.6% contamination, and the GC content was 67% (Table 1). It did not encode genes associated with carbon fixation, N_2_ fixation, Fe(II) oxidation, or dissimilatory sulfur cycling. MAG 1 was recovered from the coassembly and was most abundant in the emergence (Fig. 2).

## DISCUSSION

### Carbon fixation.

Lithotrophic carbon fixation can be a significant source of primary productivity in AMD ecosystems (44). At Cabin Branch, *Ferrovum* spp. are abundant, ranging from 5 to 33%, and likely contribute to primary productivity (33). Here, we recovered 7 MAGs that encode the genes necessary for carbon fixation. These autotrophs include those that are closely related to known lithoautotrophic organisms, including *Gallionella* (MAGs 25 and 26) and other *Burkholderiales* (e.g., MAGs 21 and 22), as well as *Actinobacteria* (e.g., MAGs 3, 4 and 5). This indicates that primary productivity in AMD sites may be driven, in part, by organisms that have not been considered in the past.

### Sulfur cycling.

Sulfur cycling is an important process in AMD ecosystems. Bioremediation may rely on dissimilatory sulfate reduction, especially in constructed wetlands (4). Dissimilatory sulfate reduction combats AMD by generating alkalinity, can lead to the formation of ferrous sulfide minerals in sediments, and decreases the concentration of soluble metals (5–11). Conversely, biological sulfur oxidation generates AMD by oxidizing sulfur in iron sulfide minerals. MAG 21 encodes homologs of *dsrA*, *dsrB*, and *aprA*, indicating that it may be capable of dissimilatory sulfate reduction, at least from APS to sulfide. Therefore, this taxon may play an important role in constructed wetland bioremediation.

AMD occurs naturally when weathering processes expose sulfide mineral-bearing rocks to oxygen-rich water. The result is the oxidation of these sulfide minerals, which produces sulfuric acid (H_2_SO_4_) and dissolved metals. Iron-sulfide minerals such as pyrite can also be oxidized by biological sulfur oxidation (45, 46). The oxidation of iron sulfide minerals may occur either at a pyrite vein or in freshly deposited sediments that are exposed to oxygen. We recovered a single MAG, MAG 22, that contains the genes necessary for sulfur oxidation from thiosulfate to sulfate. This taxon is unlikely to cause the oxidation of sulfide-bearing minerals but may play a role in aqueous sulfur cycling in the environment.

### Nitrogen fixation.

Common Fe(II)-oxidizing organisms in AMD environments, such as Ferrovum myxofaciens, are capable of nitrogen fixation (30–33, 47) and may provide fixed nitrogen to AMD communities. Four of the MAGs recovered here (MAGs 13, 20, 21, and 27) contain the genes necessary for nitrogen fixation. These organisms may serve as a source of bioavailable nitrogen in AMD ecosystems and, in so doing, increase the productivity of their communities.

### Fe(II) oxidation.

Fe(II) oxidation is a key process in AMD environments for bioremediation and as a source of energy to drive primary productivity. Indeed, our previous analyses recovered multiple abundant *Ferrovum* MAGs whose genomes are consistent with carbon fixation coupled to Fe oxidation (33). Here, we identified 9 MAGs that encoded homologs of the Cyc2 protein involved in Fe(II) oxidation (37) (Files S2 and S5). These MAGs were recovered across sample sites that range in dissolved oxygen and Fe(II) concentration and are present at relative abundances that suggest key roles in community function (Fig. 1 and 2). Seven of the MAGs that encode Cyc2 belong to proteobacterial lineages with other known Fe oxidizers. Additionally, MAGs within the recently discovered phyla *Elusimicrobia* (MAG 13) and *Eremiobacterota* (MAG 15) appear to encode Cyc2 (File S2). These taxa have not previously been recognized as Fe(II) oxidizers, but the recovery of Cyc2 in these MAGs further expands our knowledge of the taxonomic diversity of Fe(II) oxidation. MAG 13 was abundant at the emergence, where oxygen and Fe(II) concentrations were 77.5 μmol/liter and 403.2 μmol/liter, respectively. MAG 15 was recovered from the retention pond (Rose Pool), where oxygen and Fe(II) concentrations were 401 μmol/liter and 11.16 μmol/liter, respectively. Rapid iron oxidation rates in low-pH AMD environments have been attributed to *Ferrovum* spp. (which are abundant in Cabin Branch [13, 37]), and thus, these taxa are of particular interest for bioremediation of Fe(II)-impacted water. However, *Ferrovum* spp. have proven difficult to culture and characterize. The recovery of multiple Cyc2-containing MAGs across oxygen and Fe(II) gradients highlights the potential for developing alternative target taxa for bioremediation of AMD-impacted waters.

### Phylogenetic relatedness and metabolism.

In the absence of characterized isolates, we often rely on phylogenetic relationships between taxa found at AMD sites and their closest cultured relatives to infer their role in biogeochemical cycling (13, 22, 48, 49). This approach leverages the use of 16S rRNA gene amplicon data, which is relatively inexpensive in terms of time and cost, at the expense of the metabolic insights inferred from expensive and time-consuming ’omics approaches or validated by culture-based approaches. This approach—inferring physiology from 16S rRNA gene sequences—can be informative for major metabolic pathways when taxa are closely related to their nearest cultured relative. For example, like Gallionella ferruginea, the metabolic potential of the *Gallionella* MAGs (MAGs 25 and 26) recovered here is consistent with chemolithoautotrophy fueled by Fe(II) oxidation. These relationships are less robust with increasing phylogenetic distance.

Inferring metabolism from16S rRNA gene sequences becomes more difficult as the number of available genomes from similar environments decreases. For example, AMD environments often host organisms within the archaeal order *Thermoplasmatales* (9, 22, 23). However, there is a paucity of *Thermoplasmatales* genomes available from AMD environments. This order also contains taxa with diverse metabolisms, including Fe(II) oxidation (24), obligate heterotrophy (25, 26), and sulfur respiration (27). The lack of genomes and culture representatives from AMD environments coupled to the physiological diversity of *Thermoplasmatales* makes it difficult to interpret the role of these archaeal AMD environments. The *Thermoplasmatales* MAG presented here appears to be a heterotroph capable of aerobic respiration.

The role of taxa affiliated with uncultivated or recently discovered phyla in biogeochemical cycling in AMD is particularly difficult to predict. Here, we presented MAGs from four such phyla—the *Dormibacterota*, the *Elusimicrobiota*, the *Eremiobacterota*, and the *Patescibacteria*. *Dormibacterota* and *Patescibacteria* are not widely reported in AMD. *Eremiobacterota* inhabit multiple mining-impacted sites, including stalactites in a mining cave (50), neutral mine drainage in Brazil (51), and AMD in the eastern United States (28). The abundances of the *Eremiobacterota* correlated with total organic carbon in an AMD site in China (71) and were recovered from Rose Pool, where dissolved organic carbon is present (188.0 μmol/liter) (33). One of the *Eremiobacterota* MAGs (MAG 15) at Cabin Branch encodes a Cyc-2 like protein that may be involved in Fe(II) oxidation. Therefore, this taxon may play an important and underappreciated role in Fe cycling in AMD environments.

*Elusimicrobiota* have also been found in AMD environments across the globe, including in Spain (50, 52), France (29), and Svalbard (53), but it is not abundant in these environments. The only cultivated taxa from this phylum are strictly anaerobic (54–56). The *Elusimicrobiota* MAG from Cabin Branch encodes genes for three terminal oxidases and likely employs aerobic respiration. The *Elusimicrobiota* MAG (MAG 13) was abundant in the emergence, where dissolved oxygen is present, albeit below saturation (77.5 μmol/liter). The MAG also contains the genes necessary for nitrogen fixation and may encode a Cyc2-like protein that it may use for Fe(II) oxidation. Therefore, it likely plays an important role in nitrogen cycling in AMD environments and may also contribute to iron cycling.

A combination of ’omics-based approaches and cultivation can increase our ability to correlate function with taxonomy from 16S rRNA amplicon studies. Here, we present high-quality MAGs from AMD sites to increase our current understanding of community composition and function. These data reveal previously unrecognized taxa that contribute to carbon, nitrogen, and Fe(II) cycling in AMD. In particular, these data underscore roles for previously uncharacterized *Gammaproteobacteria* in Fe(II) oxidation in addition to uncultivated or recently discovered phyla, the prevalence of *Actinobacteria* across AMD sites that range in oxygen and Fe(II) concentration, and taxa with high relative abundance whose function remains unclear. These data provide a framework to assist in culturing taxa of interest as well as additional target organisms for AMD bioremediation strategies.

## MATERIALS AND METHODS

### Site location.

Cabin Branch is an acid mine drainage site in the Daniel Boone National Forest in Kentucky, near the border with Tennessee. Detailed geochemistry, isotopic analyses, ^13^C uptake experimental results, and the results of community analysis using16S rRNA gene amplicons were described previously (13, 33). Briefly, groundwater at Cabin Branch emerges at pH 2.90 and flows down a limestone-lined channel with a pH of 2.92 (installed as a passive remediation strategy) and enters a pool (Rose Pool) which has a pH of 2.97. Dissolved oxygen increases down the drainage site (77.5 μmol/liter at the emergence, 401 μmol/liter in Rose Pool), and the Fe(II) concentration is 403 μmol/liter at the emergence, 882.0 μmol/liter in the limestone-lined channel, and 11.16 μmol/liter in Rose Pool (33). The concentration of dissolved inorganic carbon was highest at the emergence (1.67 mmol/liter) and lowest (0.30 mmol/liter) at Rose Pool. The concentration of dissolved organic carbon was low at all sites (36.8 to 44.2 μmol/liter). The concentration of NH_4_(T) was highest at the outflow site (50.0 μmol/liter) and lowest at Rose Pool (10.7 μmol/liter). The concentration of P was highest at the emergence (9.55 μmol/liter) and decreased to below detection limits at Rose Pool.

The microbial communities at Cabin Branch have been studied and described previously (13, 33). The communities are dominated by the Fe(II)-oxidizing genus *Ferrovum*. Lithotrophic metabolisms, likely Fe(II) oxidation, drive primary productivity, and *Ferrovum* spp. encode the enzymes necessary for nitrogen fixation and thus are a potential source of fixed nitrogen (13, 33). Methods for sample collection, DNA extraction and sequencing, and metagenome assembly and binning were described previously (33), and we include brief descriptions below (13, 37). Methods for sample collection, DNA extraction and sequencing, and metagenome assembly and binning were described previously (33), and we include brief descriptions below.

### Molecular analyses.

**Sample collection, DNA extraction, and sequencing.** Triplicate samples from each site were collected for DNA extraction and were flash-frozen and stored at –80°C until processed. DNA was extracted from each replicate sample using a DNeasy PowerSoil kit (Qiagen, Carlsbad, CA, USA) and quantified using a Qubit 3.0 fluorometer (Invitrogen, Burlington, ON, Canada). Extractions were pooled and submitted to the University of Minnesota Genomics Center for metagenomic sequencing and sequenced using HiSeq 2500 high-output 2 × 125-bp chemistry. Three samples were sequenced per lane.

**Metagenomic analysis.** Trimmed, quality-controlled sequences were assembled using MegaHit v. 1.0.6 (57) using standard parameters except for the minimum contig length, which was set at 1,000 bp. Reads were mapped to the assembly using bowtie2 v. 1.2.2 (58), and the depth was calculated using the jgi_summarize_bam_contig_depths command in Anvi’o v. 6.1 (59). Binning was performed in MetaBAT v. 2.12.1 using default parameters (60) and CheckM v. 1.0.7 was used to determine bin completeness (61). Bins that were >70% complete with <3% contamination were selected for further analysis. The average nucleotide identity (ANI) across the surviving bins was calculated using anvi-compute-ani in Anvi’o v. 6.1, which uses the PyANI algorithm to compute ANI (59, 62). Bins that shared >99% ANI across the genome were considered to be the same taxon. For each taxon, the bin with the highest completion was selected for further analysis. Bins were uploaded to KBASE and annotated using the “annotate assembly and re-annotate genomes with prokka” app v. 1.12 (63) and classified using the GTDB app (64–67).

Single-copy, ribosomal protein sequences from Campbell et al. (68) were retrieved from the MAGs and reference genomes, concatenated, and aligned in Anvi’o (69). Anvi’o uses MUSCLE to align the concatenated sequences (70). Maximum likelihood trees were constructed using RAxML-HPC2 v. 8.2.12 on XSEDE in the CIPRES Science Gateway using standard parameters as follows: 100 bootstrap iterations, a Protein CAT model, DAYHOFF protein substitution matrix, and no correction for ascertainment bias (71, 72). Trees were visualized and rooted in the Interactive Tree of Life (73). A Newick-formatted tree file is available in the supplemental material (File S1).

The relative abundance of each MAG was determined by mapping reads from each metagenome against each MAG using BBMap (74). The pileup tool within BBMap was used to summarize mapped reads, and the relative abundance was calculated from the total number of mapped reads divided by the total number of reads in the metagenome (75). Unmapped reads and reads mapping to more than one region were removed using SAMtools (76) prior to pileup.

Metabolic pathways for carbon, nitrogen, and sulfur cycling were predicted in each MAG using METABOLIC v. 3.0 (77). Each MAG was also analyzed using DRAM v 1.06, which generates and summarizes gene annotation data from multiple databases (78).

Cyc2 genes were identified using BLAST to identify genes homologous to the Cyc2-like cytochrome *c* involved in Fe(II) oxidation (36–38). A BLAST database was constructed using the Cyc2 retrieved previously (37), and the search was performed using an E value of 1E-5. To ensure that the sequences retrieved with the BLAST search were homologous to the Cyc2-like protein involved in Fe(II) oxidation, retrieved sequences and those described previously (37) were aligned in MAFFT v. 7.471, portions of the alignment that contained >50% gaps were removed using TrimAI v. 1.2.59, the best model was selected using ModelTest-NG on XSEDE v. 0.1.5, and a maximum likelihood tree was constructed as described above except using the LG + G4 model selected by ModelTest-NG. The Cyc2 tree is displayed in File S2 and available as a Newick-formatted tree in File S3.

### Data availability.

Quality-controlled, unassembled, metagenomic data are available in the NCBI Sequence Read Archive under accession numbers SRR9677580 to SRR9677585. The metagenome-assembled genomes used for analysis are also available in NCBI under accession numbers SAMN14771053 to SAMN14771081. Metagenome assembly and mapping statistics are provided in File S4.
