# Supplementary material for: Metagenome-Assembled Genomes of Novel Taxa from an Acid Mine Drainage Environment
Source: Appl Environ Microbiol. 2021 Aug 11;87(17):e00772-21. doi: 10.1128/AEM.00772-21 (PMC8357290; doi:10.1128/AEM.00772-21)

File S1. Newick-formatted, maximum likelihood tree of concatenated ribosomal proteins for all MAGs.

((2861560136:0.33478129258414240921,2786546906:0.32378900267443438965):0.05954145348368599516[100],
(((Cabin_C_42:0.47113381195184611538,(2786546856:0.37677510144137155157,
(2654587719:0.26968924114888448429,2582581009:0.27372831273331776902):0.08461722648001748781[100]):0.115785564937
34182276[100]):0.69288825318377189610[100],(((((2502171154:0.83998133208396319471,
((2728369529:0.99134292948472413354,2770939392:0.81356328979048087024):0.32491368721473490311[100],Cabin_Combine
d_108:1.13868032782376871204):0.19324487291761047181[86]):2.85677279068188738620[100],
((A_2698536868:0.84264447619096227715,
(2548877146:0.94521287074887772039,Cabin_C_3:1.33390623936023811069):0.21812619980879963122[100]):0.1912300073176
4425164[100],2713897420:1.56503122997281063356):0.17637187995363518001[100]):0.09251994557119053164[89],
((((Cabin_B_29:0.26644226678525928520,2786546843:0.27368443787720309057):0.20207136459870628231[100],2523533552:0.
34423598719729053830):0.37080295738747587331[100],((A_2824080494:0.51676623044033731524,
(Bog_877_GCA_003164475:0.26091323037351027425,Cabin_C_37:0.21932991398951987017):0.50797484031643858060[100]):0.
29742299737060240439[100],(A_2667527204:0.48402502512092571152,(A_2734482170:0.27599509391776860801,
((A_2616644938:0.20692033619333727312,A_2667527203:0.17980700593396048426):0.06945639881828864770[100],
(Cabin_C_38:0.01666034019892964971,Cabin_B_6:0.01447067152765537691):0.37463405712777653189[100]):0.0680355894185
3710129[100]):0.24252261958248352847[100]):0.37555748459109961068[100]):0.07259890747746851336[85]):0.04894572602192
991118[63],
((((((Cabin_C_30:0.03722192997896398708,Cabin_A_14:0.04249189179673062283):0.17471996141357848398[100],Cabin_A_25:0.
19317153657922050969):0.19904730336147136160[100],((A_644736322:0.39014943796813555954,
(A_2585428130:0.00028498506752636414,A_2571042004:0.00061315081860729081):0.31410192296258931943[100]):0.2640820
0944072229843[100],A_2582580746:0.36383516235555130036):0.06457719183357510384[88]):0.07515021945793115554[100],
((Cabin_B_5:0.01925815441669264175,A_2585428127:0.00484118629778580289):0.43797523698882101506[100],Cabin_Combin
ed_109:0.39272504464096241739):0.07483922302399022286[100]):0.10158417504872074844[100],
(Cabin_Combined_90:0.49438038823911906183,
(A_2728369701:0.38411967717547518264,A_2698536723:0.33146834938439362350):0.05180019918080989993[71]):0.06604929
650644027206[99]):0.08667547608478400056[100],
((Cabin_C_65:0.15885901449134157137,Cabin_Combined_60:0.11493237728818904431):0.07824909441065044935[100],Cabin_
Combined_56:0.17584259340639302183):0.42798907409304309812[100]):0.56487586780841403389[100]):0.06941942098380753
101[93]):0.08403626119785188864[89],(Cabin_A_27:1.08235553270405282511,(2519103101:1.07062313883531623482,
((Cabin_B_32:0.10232670949618447032,(2861273687:0.07151281608166847048,(2582581264:0.06244586642722155939,
(2531839705:0.00612616613095820321,2556921625:0.00410795503045782804):0.05788572568216790448[100]):0.061838536328
45612291[100]):0.03946906655428703697[95]):0.75518375024484352132[100],(((Cabin_B_11:0.59661217318847736824,
((2816332403:0.07812045907034240178,(2816332401:0.05524708553724436144,(2816332407:0.00011894380821429988,
(2816332376:0.00436870245263414319,2778261609:0.01242134484830379262):0.00014421302131421891[96]):0.0483397897054
1136233[100]):0.05247551988803456080[100]):0.26131740687823901137[100],
(Cabin_B_8:0.32627820464504403430,2710723679:0.41298765023484335090):0.13038224895493233380[100]):0.1264722373350

2283016[100]):0.05394636680951263308[100],(Cabin_C_28:0.28542224678789196446,
(2861661002:0.13508541529851131324,2721755797:0.14941858851683287313):0.15077197337836564794[100]):0.217409755577
97623252[100]):0.08405371325503135926[100],(Cabin_B_30:0.46528628531465687779,
(((2671181060:0.07752941691043371830,2690315850:0.06770428583400804612):0.15954233164126399580[100],
(Cabin_Combined_34:0.16041878582098476058,
(2636416104:0.13651258948336095211,Cabin_B_25:0.12429569713549222520):0.14110227602829605864[100]):0.053131234667
07372206[100]):0.15841265595532497135[100],
((2775506857:0.21170869560508992491,Cabin_Combined_18:0.29989556046015652946):0.03864924857023468302[97],
(((2788499943:0.10315824775872531327,2721755281:0.06462796300899471824):0.08736465812708535394[100],2574180436:0.1
4071200326668767411):0.03337900984730846066[100],(2264867016:0.32949573201659687616,
(Cabin_B_27:0.05025227790847579951,Cabin_Combined_37:0.05329906895563727570):0.07152260337288685044[100]):0.07300
344900357208511[100]):0.15229487888882289881[100]):0.04728959129457684096[90]):0.06814612784004676904[100]):0.175528
71593057881028[100]):0.27030237339709062283[100]):0.15531759165978900605[100]):0.06984835868270979076[44]):0.0558935
7566935729965[92]):0.06417625865143418429[98],
((Palsa_948:0.36750060733805933744,Cabin_Combined_77:0.41489761695499099980):0.73042367848721312384[100],
(A_2786546774:0.49780554916139085897,(A_2751185594:0.52495746505756890610,
(Cabin_Combined_21:0.14325063197557139083,Cabin_A_35:0.14972430480617449078):0.39173700755993784428[100]):0.13442
283327740697785[100]):0.49336107191865374855[100]):0.11863133871677979114[97]):0.11200367715514768230[97]):0.5820320
6565469656208[100],
(Cabin_C_50:0.25448220724524217573,Cabin_C_9:0.26621361647820163121):0.34130725666310479660[100]):0.2798748840714
2835367[100],2823575546:0.41956222297473000360);

File S2. Maximum likelihood tree of Cyc2-like proteins retrieved from Cabin Branch MAGs and reference sequences.

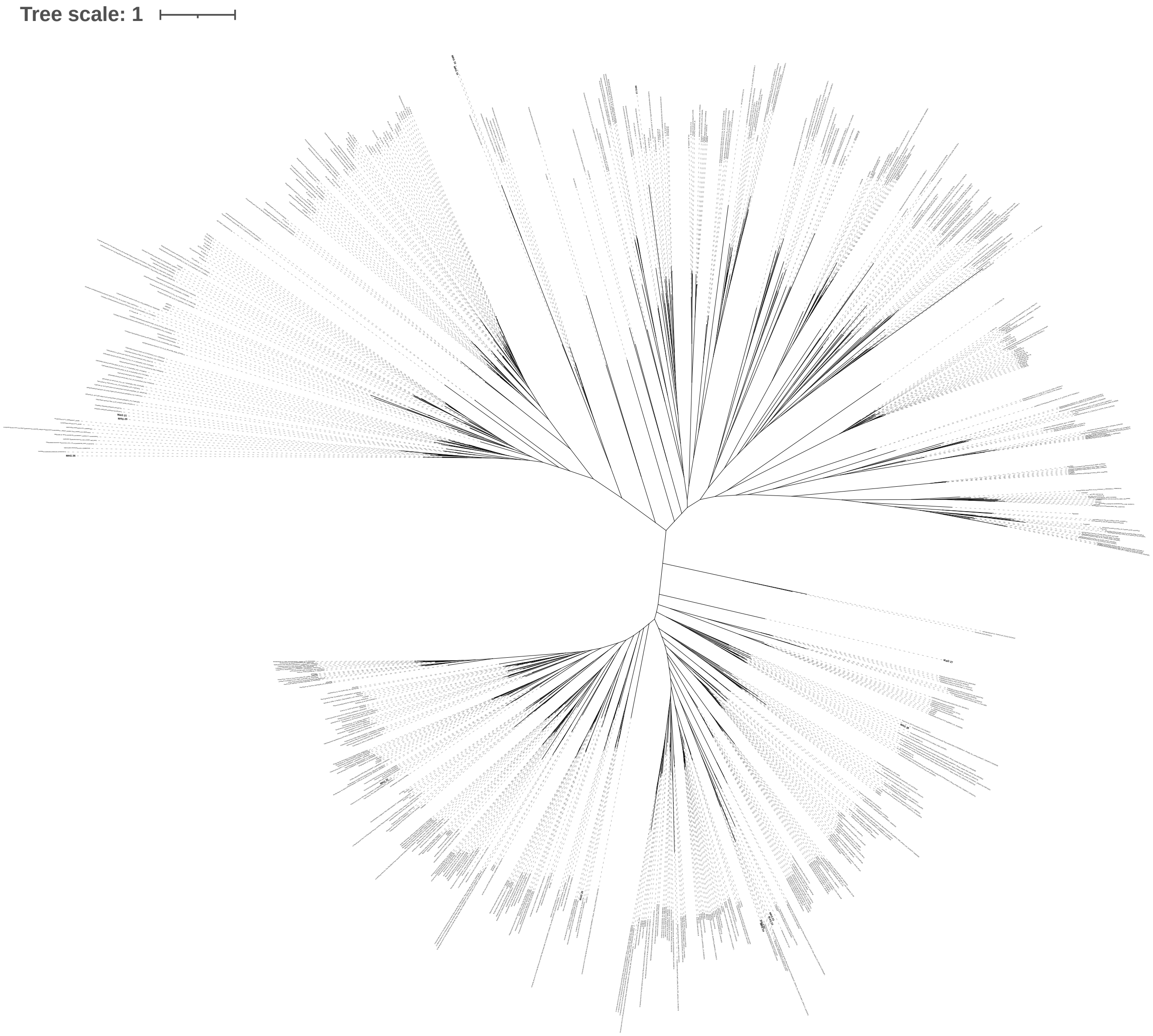

File S3. Newick-formatted, maximum likelihood tree of Cyc2-like proteins retrieved from Cabin Branch MAGs and reference sequences.

(CabinCombined37_k99_2722425:0.16442750696863159265,(646687912:0.26133854918300791770,
(((GAAA018N21:0.19261997997076021893,
(((Famni_OYT1:0.10708233100717431951,Ferriph_R1:0.13710911417416324354):0.23558380406445084132[100],646687913:0.19
327094065402661283):0.04226146920110841754[30],2582974111:0.25416559274143035729):0.04074566849909137811[26]):0.03
926637256764456407[32],
(2713871873:0.22967455621068089133,Gcapsi_ES2:0.19450913124649488184):0.05235017559506248419[46]):0.1233090685844
9409980[100],((Cabin_B27_k99_2337392:0.12765714611513054311,(CabinCombined_18_k99_170614:0.04092495877747870381,
(KXS32103.1:0.07823050579029082463,KXS32176:0.08799188595270793223):0.03402612972634656147[84]):0.10009797364538
589037[100]):0.22165762146859210868[100],
((OGT00877.1:0.17544206552248542419,2700913626:0.11557252819688829115):0.32733640190050888874[100],
(((3132051297:0.94415483410902556471,
((2709538937:0.00000100000050002909,OGT19314:0.00000100000050002909):0.79269633335812161867[100],
((((((OIO71009.1:0.19528384702122153493,
(2529064164:0.00000100000050002909,C09_ZOTU6:0.00000100000050002909):0.22062326739778773121[100]):0.128744592674
39036791[100],312260537:0.21730410428685845159):0.09427761598376294128[87],3121321203:0.37580680553874296068):0.05
03467442525551423 0[24],((31220339:0.21645710097178821885,(CG1_Bin2b:0.21708574170919905932,
((2651849445:0.00000100000050002909,Mf_PV1:0.00000100000050002909):0.04696377417073034632[100],Mf_M34:0.05541092
676163824476):0.11301714519635162881[100]):0.05508852022445420715[63]):0.06922982082804403359[50],
((((((3121131163:0.18603503672368515431,312253196:0.09939400362132529143):0.02759215579651209602[56],3121841:0.1040
9030551633409456):0.08971628140919683803[100],312123298:0.12943002267429432051):0.03236147805325044480[33],
(((312149188:0.10723112119966918265,312212459:0.20528449797291506429):0.02536853288702993481[21],
(312160971:0.08022028091747199219,312198806:0.09047169949696776126):0.10969710603575154317[100]):0.03501778518014
903724[18],(((3122532194:0.08023194248497278469,
(3122451495:0.00000100000050002909,3122221137:0.00000100000050002909):0.13498093153219339890[100]):0.036960000959
59429271[65],(3121461293:0.11491863030025527281,
((312184425:0.00000100000050002909,3122141194:0.00000100000050002909):0.01428576073396483140[100],
(((31222641:0.00000100000050002909,312188843:0.00000100000050002909):0.00000100000050002909[34],M07_ZOTU10:0.000
00100000050002909):0.03130174829883584919[100],3122001323:0.01306889120520269253):0.00586803569296925811[61]):0.11
918633472555402453[100]):0.15836162866549993722[100]):0.02730634723487397955[51],312113851:0.24004542124982047868)
:0.08980600842387463645[99]):0.01212288623376850220[4]):0.02427002186671224637[2],
(312146852:0.13670388844363917658,
(312184947:0.03067784196216202797,3122001971:0.00278171675372001874):0.12520817120112184839[100]):0.1054967821927
3050939[100]):0.03102506486426658688[18],(312173451:0.18471387480845857798,(312156880:0.06067688906787038067,
(((3121291632:0.00923929735567582087,312235449:0.01721989498404580959):0.09791146687767381118[100],312108363:0.072
79085485830125157):0.02219565091116462024[45],3121281675:0.07723869924578904977):0.02806217991579492907[60]):0.181

31284264252608973[100]):0.18936827635293135286[100]):0.04049949643945624511[57]):0.13161554172636388826[93]):0.10586
425419226255795[87],(((Ma_CP5:0.09515395081223579354,
(072659392:0.15981885477455645694,Mf_CP8:0.13039811133111847696):0.03760439487701044969[77]):0.07506135531834054
464[99],(Mf_EKFM39:0.14512937877523407471,
(OIO71828:0.09313744718370700615,3122201895:0.17682969708507623174):0.06605535734102097045[99]):0.04975106397880
647846[83]):0.15696134728372387879[100],
(((2525813086:0.01854135958577683360,2525849410:0.02882978580331853335):0.17947251277924533563[100],
(312109836:0.17005986042060122765,
(Gb_TAG1:0.24697734091637787990,2617305037:0.21771545188104710067):0.11539990046745804420[92]):0.072081646374844
68229[75]):0.09272787222786540995[100],
(312223445:0.26028036855260017202,CG1_Bin2a:0.22734110517203712232):0.15814056118988778499[100]):0.1024208389925
2431289[99]):0.10647651962340287657[97]):0.24404337686401755825[100],
((2559026979:0.00000100000050002909,2572237054:0.00241609710174755686):0.23852615941435884039[100],
(312241922:0.10600803381095316724,CG1_Bin1:0.17987858669285006208):0.27065524790903211505[100]):0.48587965868850
147011[100]):0.25183066638771411938[100],
(((((642750112:0.44357098568769681712,OSS41111:0.34474140931798730181):0.54370165571184048137[100],
(Cabin_B8_k99_569411:0.72666219563154332839,
((((((3287272378:0.49215077830355974386,2583030689:0.32715133451411604337):0.02664944129600335318[7],
((2583018665:0.27291270613495510755,
((2595244049:0.14471709758361450682,2582970236:0.08844835401027466293):0.09296330500820471077[100],2583041736:0.1
7154631873326686708):0.04787135766178732960[68]):0.14584141182468851605[100],
((264772531:0.08337003239346206518,32886269:0.04644459095803452758):0.14517514645781964822[100],
(2583012401:0.20728997580580943438,2504619835:0.19267689876370833191):0.05149614746655013092[66]):0.1912218694033
3779637[100]):0.05996863217812439112[62]):0.07569266857070809651[41],ODT02650:0.58410180202771910807):0.0442034722
9316851090[43],
((2595265659:0.39798760940775490536,Cabin_C28_k99_144128:0.23784086797892936560):0.05618324395730658893[46],ODU9
9277:0.29329512237831489863):0.16830463600918202371[99]):0.07128493222878130864[31],
((((2709537374:0.52412685123072588755,(2722291371:0.33601271411823419033,
((((051426997:0.03456744949698673658,020563078:0.04818753955341305295):0.17934781491473889004[100],077729706:0.215
55264991792663642):0.04664975072304388543[73],
(2508503523:0.40901830525085602908,2619780814:0.26174342209817058613):0.05475057357344394193[72]):0.1000649824610
2007998[100],(ODU11348.1:0.35359838784770414311,
(2518923468:0.00235032821803971134,2651442434:0.01227033709422508528):0.25799878864600284123[100]):0.133370560675
71814721[100]):0.13283557015656369260[99]):0.07709129048001080176[59]):0.05855687985973308946[60],
(2621211688:0.21455636054794618994,2617182549:0.31390130441198682565):0.21490079811045698088[100]):0.050328332687
20455361[19],
(((2714739554:0.15176784138838095295,2596953190:0.13901194848390402425):0.10523091393891940981[93],2647562834:0.14
071511108711781679):0.33391731168374638727[100],
(((((CCA88085:0.01031720040744881980,2688987667:0.00655295699711607674):0.03592659114489734490[100],

(048934802:0.06551567280684300609,((2538680252:0.01053586756414009312,
(2570846834:0.00000100000050002909,2655903392:0.00209288837867616529):0.01501981000601782489[100]):0.030734579276
34626330[88],
(2549744039:0.00626000916849667622,2652266820:0.01069477679521788765):0.03697048965715678953[87]):0.0097519763824
4680760[26]):0.01492023082394033397[30]):0.06982396789800625336[81],
((Cbasile_B8:0.06191900772647021545,Cbasi_OR16:0.03310722473338014588):0.05716576573671581368[96],2609799710:0.199
25700334897708532):0.06372937501744653510[63]):0.49598621184359104053[100],((Psgul_NH8B:0.12600058436955330343,
((2628330780:0.00000100000050002909,KIA78966:0.00217075177218261587):0.05206076705053384879[100],
(2714105722:0.05195324610720177677,(((2714091736:0.00000100000050002909,
(OBU84774:0.00244027420426232503,2632595325:0.00181217506411332366):0.00036001597897518622[20]):0.00434205894714
334920[64],2714096225:0.00000100000050002909):0.07175617919176313642[100],
(2721279830:0.00214238334426407025,2632161144:0.00000100000050002909):0.06906165522312741889[100]):0.069651057714
11263247[97]):0.03320935682227527108[62]):0.16759922448836478415[100]):0.27217633569804516869[100],
((2582856064:0.10890386331200871406,646832890:0.15395510533142398035):0.71355786819157207557[100],
(((Burk_GJE10:0.77261546613819953588,(CabinCombined34_k99_2589288_2:0.24578613684264993822,
(((2595257053:0.15523636625544920409,
(Fer_PNJ185:0.04436827112418280372,Ferro_JA12:0.05960878496695099643):0.25072390641090730456[100]):0.0856826621058
6879261[100],CabinCombined34_k99_2589288:0.06642955355172151799):0.08995659359460135562[100],
((Ferrov_Z31:0.03255410049819578572,Femyxo_P3G:0.02470551682736791752):0.06370054410568799785[84],
(CabinCombined34_k99_1422742:0.00000100000050002909,Cabin_B25_k99_1560804:0.03460276370289909476):0.02461690440
280966258[74]):0.13544594586839173167[100]):0.13764072344907724021[99]):0.24467255437827994946[99]):0.14975533956204
625796[91],(2642723526:0.11218085330045772519,
(640470699:0.00000100000050002909,071466569:0.00432173270954971673):0.03372060731832234037[96]):0.544459602486902
72731[100]):0.16088821439280417969[68],((((081427325:0.00986293068201514293,
(2504722960:0.00719378100282372281,2687112497:0.01747942347562966153):0.03165372160328568801[70]):0.1315574192888
0512748[100],077562542:0.13678357215537578773):0.20608089293586950852[100],
((OAJ53554:0.04052787285274624812,2671593425:0.05635094338875336339):0.07959131282176878930[100],
((2600793029:0.00000100000050002909,051391183:0.00407059180886855040):0.09259073265786248175[100],
((2516023047:0.03889344867846506598,083245405:0.02253154627393319509):0.05523242309055080568[100],
(((071334897:0.03458428978329770864,(AOJ37486:0.03093325497859916035,
((059482638:0.00211339870994397758,2598452010:0.00417657881445470096):0.03391135063545436951[100],
(059807244:0.01233097346476081692,059467121:0.00938731690912585071):0.01498351277763490010[95]):0.011704127084179
10112[48]):0.01799345557137687832[55]):0.03415469523114703781[95],
((080408032:0.00677949924858645830,KVP23108:0.00000100000050002909):0.03155250758512642001[95],
((084904304:0.02076834655518344785,2552801158:0.02698371919605440511):0.01346020517494774665[83],
(053077704:0.03830444891804649782,GAU02319:0.03889871293563465504):0.04030329119628803247[93]):0.008383142657881
95345[60]):0.03186882357457661297[72]):0.01938502079249187354[43],
((059759890:0.02032903064683731428,KVV32910:0.00740549806573765831):0.03465515900411927919[99],
(AOI91739:0.03016591889299079232,2648568615:0.03547046407051600986):0.03249336534259256865[88]):0.014608088446656

20679[46]):0.03809790572491315169[84]):0.05801540265006745883[96]):0.06561851029704250660[96]):0.1352054295128769789
7[100]):0.12908478206234602625[98],((((((2582911414:0.16245758938348814349,
((059422050:0.00217455108366936684,GAO36401:0.00000100000050002909):0.01283633057251494514[100],
(051338760:0.00000100000050002909,2533686707:0.00000100000050002909):0.02106740154405522397[100]):0.1654080537280
0362893[100]):0.06365338306679022007[85],(Rferr_T118:0.15660614582298096331,(2583035179:0.49220238589128378903,
(2582925493:0.08172766088217846281,
(2582927270:0.00000100000050002909,2582928415:0.00000100000050002909):0.04305423794426802703[100]):0.169474526606
06834862[100]):0.05890145613073349135[48]):0.05824921639290041309[55]):0.04236522016675968449[50],2510552914:0.26645
188483447918015):0.17571261356013603838[100],2515877855:0.31667040818922514989):0.05332409035371572792[35],
(AJP49544:0.00000100000050002909,2595015798:0.00253351228592554791):0.28390912452827621326[100]):0.0323154388734
9853927[20],((Thiodenitr:0.05876466329931252786,(ODV00163:0.00000100000050002909,
(2515446894:0.00928996952343378278,2628007049:0.00478946284772270928):0.00236369028160581147[28]):0.0739925384873
6646322[100]):0.33819854803564564083[100],
((2651322974:0.09671109599462278383,2510566723:0.57349504594846067995):0.37845802128207928217[100],
((2628000674:0.23317676780983451201,2586349735:0.09977249625211434547):0.03164867028479838307[70],
((2609801455:0.01195157299329890205,
(2609814799:0.01127772465471716355,2609802997:0.01213336293805644325):0.00701009604962102555[57]):0.0201025808693
8494088[95],
(2674585791:0.04769377308588862441,2609810712:0.05113034114969626509):0.01565451488030554852[77]):0.0577793880172
8697837[95]):0.15267291489180057074[100]):0.04387407672357499649[32]):0.05745110865092916547[16]):0.0567824469707083
7510[13]):0.05502650565539405486[31]):0.07451766253752480085[73]):0.07136779212833657682[56]):0.02558894048013494668
[31]):0.05947014655877623512[16],(((2566048413:0.21375671527298437113,
(2609552291:0.08003660197696020606,2690600517:0.09100409228669698036):0.21266758538271227086[100]):0.163860046087
40953326[100],((059615940:0.08258975568510053267,
(2632122048:0.02030483365513175917,082727632:0.03950971249484493131):0.03620561357173185391[97]):0.05741763089088
838812[95],(2649142128:0.14878271422496563980,(((2722725131:0.04890862241814834116,
(2552778636:0.00000100000050002909,051003599:0.00219224614560943219):0.06225647829047814552[100]):0.0638523508579
9410848[97],
(052495082:0.00000100000050002909,2723150964:0.00228459108569022086):0.08983439441619125998[100]):0.0396677311739
3353199[69],(2551938518:0.17735240440641741877,
((2699687893:0.02604902181125134594,Ralpickett:0.01593310636047523196):0.03576349721136882348[99],
(Ralso_FQY4:0.03123144153668446291,
(2655899363:0.03423752948232565430,CCA86568:0.03337421808831163295):0.02647069542199974659[87]):0.01293847606981
082810[71]):0.08373547267171578545[99]):0.04614384220967439332[67]):0.05074633721304797757[67]):0.033257930775314976
93[35]):0.19034046593363626587[100]):0.04818810132933971230[36],
((2519813614:0.00802468699169294614,2545744318:0.01437442042397679177):0.40920575107713408336[100],2582855356:0.3
5546597613269326210):0.08788489264284427005[57]):0.04897404690239551966[33]):0.05475730867100211258[32]):0.05994246
014441664133[35]):0.06672593547081384635[14],((((((2597913977:0.35120743142430632666,
(((Rhodop_ATH:0.00000100000050002909,

(Rhodop_S55:0.0022062981776711232 4,Rhodo_WS17:0.00000100000050002909):0.0044148615377927911 6[87]):0.08118130385
980285024[100],
(2671915913:0.00000100000050002909,637963819:0.0065335679629010944 7):0.0908833403374998127 8[100]):0.3805780806177
5183495[100],(Bra_ORS278:0.1524261628898003206 9,(2653887682:0.1687880106038638916 6,
((2597911083:0.1047324983384701757 6,((2636530532:0.1379584449504258347 7,
((2700928529:0.00000100000050002909,074278395:0.0022698149774705036 2):0.0080450420461883678 8[95],
(2694655162:0.0248073969035914089 5,
(074278193:0.0022533191320198630 8,2700926766:0.00000100000050002909):0.0057730761807951074 3[76]):0.00779019899398
117333[56]):0.0615188547880130137 7[100]):0.1461844027776434129 5[100],2698418960:0.0392490085195072654 9):0.032604335
63458768613[30]):0.0300964046426313719 4[14],2514440204:0.0891129319874868486 3):0.0321347226344569636 4[13]):0.046561
11359563275959[58]):0.1120481622955982886 6[99]):0.1245476842711310677 7[99]):0.1248255092730383153 4[100],
((2676342706:0.0816638388671377391 0,2510255561:0.1327993218554581467 1):0.1287735607573595453 3[100],085771681:0.33
48999353404404910 6):0.1935102709616840777 1[100]):0.1186817299690917093 5[98],(((2523410377:0.4375467201700139474 6,
(052833050:0.0150765315591076913 5,044591466:0.0118423994804639568 0):0.5550406511170720147 4[100]):0.09092413969342
018343[36],((2595262768:0.1208732300028281248 0,
(2582978841:0.1160577404594331507 6,2582897580:0.1548719348111894100 8):0.0842030844573525977 2[60]):0.5507387540380
7370787[100],((2517553761:0.2914302670159278907 4,(SJM94235:0.6106025248030225727 2,
((2636912291:0.0723992103551473614 7,2588205070:0.0430378261895002570 1):0.0758298220027770197 8[100],
((2676386204:0.1925633119076429478 1,((651002515:0.0270584305846303246 9,((2671671299:0.00000100000050002909,
(OQW81150:0.0041655936885239908 5,2727726885:0.0041855200115509822 6):0.0062310920135128459 3[86]):0.0607759443473
4440165[100],OQW42842:0.0723695350821716015 8):0.0598291622946445639 2[100]):0.1293399234498890937 3[100],651003225:
0.1955392959178437750 6):0.0439638423246725557 1[56]):0.0387215713216845641 7[33],((OJY08912:0.1286110580027881034 0,
(2671657233:0.0020747336182951872 3,2671445702:0.00000100000050002909):0.1022839178134626325 6[100]):0.093500017273
65980786[100],2636913632:0.1231870517142754500 9):0.0249424614250986324 8[23]):0.0421637619607963601 3[44]):0.14012642
738970129686[98]):0.1027500336124484159 3[78]):0.0419891076439518548 5[24],(2595102826:0.3755073285577369968 1,
((2677968525:0.1800332572720742541 1,((2574134454:0.00000100000050002909,
(082885624:0.00000100000050002909,083386236:0.00000100000050002909):0.0022388683802745329 8[92]):0.090571810118382
69513[100],(OAI29429:0.0779107418321586531 2,
(2516190406:0.0443074668295329668 3,OAI09603:0.0446192958616098006 6):0.1357859689564694383 2[100]):0.04856923786932
452330[92]):0.1765385915185372356 4[100]):0.1315421316634795001 5[100],(2585139763:0.2284989374547572171 8,
(2516960547:0.2107061455858061371 8,((2582885061:0.1603490764162706916 4,(648843130:0.0165139729949555016 9,
(2517428901:0.0303585220052909610 1,
(2582536956:0.0310264089528248000 7,2522717648:0.0236359047543854526 6):0.0132175049814897607 0[90]):0.0227708398336
5240510[100]):0.1193574521086659540 0[100]):0.0519692686394716135 7[90],2634877808:0.1155209054514899219 6):0.05231287
505914578723[88]):0.1072798577289149069 0[98]):0.0788510107316563874 1[97]):0.0315851853987875394 5[51]):0.031449654475
61526295[33]):0.1013431934770147591 1[75]):0.0698409032938754753 6[79]):0.0534418339482922513 7[26],
((((OJU07810.1:0.3103012823893036564 7,2583043224:0.2280037045308671295 2):0.1494736106035221001 6[100],
(((067617120:0.1526368735926998121 4,2523703545:0.0938166964834693528 6):0.0652523768027803152 7[69],067744063:0.1311
5728363358603037):0.1260366959096724248 6[100],

((052223625:0.06372856730878435905,067734630:0.12783839350242862110):0.13797973385420672643[100],067906227:0.1531
1880713110151420):0.06884050788918641417[95]):0.21724105120810174863[100]):0.11957487700761487703[96],
(048860060:0.22395052761179293221,
((2685829211:0.30680442386464823468,2685831528:0.19193298187446961034):0.12077372658106712289[83],ODU61985:0.255
29938441130434201):0.05126076162189777130[36]):0.33151973889788627359[100]):0.08551132701045717488[68],
(((((2521804426:0.00693249201993173698,2527007432:0.01139973244060040955):0.16590872708398504698[100],2616681780:0.
25051640766472399457):0.20861738343281266417[100],2649615031:0.43597323000008664229):0.04222921534035611879[23],
(((2515877853:0.41690692909904697450,(SBT06972:0.18885797373472829053,((2563613793:0.16248784016781805950,
(637680051:0.11689042189828563467,2506671179:0.07287355756649592509):0.07839969979301199265[98]):0.06037547351530
106465[87],2508501393:0.22938526025957212129):0.05010829849230831468[94]):0.14240358627012206227[100]):0.0856272867
4520503347[41],((646831074:0.15820931164787579992,
(2585895491:0.07607325180152649247,649772731:0.13917982142782991950):0.04281765588833947450[73]):0.21573511438771
364457[100],(2502379653:0.38228085528256283698,
(083903159:0.00227291710441901223,2563611812:0.00000100000050002909):0.38040011747748941895[100]):0.0950381183962
5322944[66]):0.01648476366257045633[15]):0.03872815392761704695[7],
(((2634019136:0.00000100000050002909,052162038:0.00218564721470527849):0.44903782405568298808[100],
((2644243628:0.00000100000050002909,056656680:0.00000100000050002909):0.30054481748474659586[100],
(((2651125062:0.12957258419981029784,2663222128:0.05914695868152067271):0.09384319983599524739[100],
(((078563083:0.00778279251972854857,007972314:0.01367145462342313732):0.01044821679742753348[74],637633170:0.01867
398073655804000):0.17883554104626195325[100],2678972498:0.19238803447793709211):0.05127417748104384082[73]):0.1158
7900881877696846[96],(2609553531:0.19031732258062880603,(((2595447474:0.12293104334570167935,
(2640590121:0.17062968597966782225,2631914223:0.12807918700854722949):0.04069433355604833852[88]):0.0451305266934
2513396[83],
((2699823700:0.06734057909124950558,2510416040:0.04980074850319154978):0.15581628651281259623[100],2558296041:0.1
6490910455629617659):0.03306829261863961089[62]):0.04659806430038417047[67],2640844523:0.16311350683256373162):0.0
2424443128864707184[32]):0.08104727459085633856[89]):0.11597763406282289578[97]):0.48922719852275403651[100]):0.0961
2772868119076608[42],(OJW46643.1:0.25411263654535082290,(2502333272:0.30457145843601501900,
(079432982:0.21911826147355889516,
((085315679:0.13049135535748709880,2529306053:0.15195089075579001903):0.10577469647875227532[99],2553574224:0.241
87784018049895973):0.05401793359839907049[59]):0.07271734417301775610[78]):0.05950216127758237161[50]):0.0797129714
3303247845[59]):0.03421966902842138447[13]):0.04746465410048002903[34]):0.06134232175024793149[60],
(GAP65883:0.39823673096422473883,(((2616226325:0.05107913951785154200,(CDW96384:0.00418605885914793193,
(651255703:0.00214790118505147178,646800198:0.00210740011963871966):0.00221392606583072473[68]):0.061039918609021
24172[100]):0.15575519921907007848[100],(Thiomo_FB6:0.21002744909307993759,
(Thiom_FBCd:0.03416285845081493827,Cabin_B30_k99_1427179:0.07904652017909011286):0.20387897103576640978[100]):0.1
3160843506463557051[100]):0.18193749162335701164[100],((2519940479:0.20290950000258839436,
(((2553029806:0.00859999311017267155,2687581785:0.01078195522682386803):0.19532484235897076830[100],
(2599169283:0.22648775253628464221,
((2506719248:0.02541784183174578340,2538844664:0.04693191883529886088):0.10040545149070081998[100],2538834093:0.1

2789946386615747009):0.08058264048757603215[100]):0.04519037113047660870[84]):0.07887909136626702900[97],
((077439889:0.03490932493935843678,077514108:0.02798314902087034595):0.15276710730457832010[100],
(038623940:0.13962992262776952046,OOG37668:0.19363824486671796232):0.02041602546489008213[58]):0.05579302298409821032[97]):0.06356203729170964434[99]):0.09160725854334196561[99],
(2522338571:0.00207104796770543869,2585805519:0.00000100000050002909):0.38659206040825339068[100]):0.15521092005211248321[100]):0.06026907464045327956[87]):0.08749682125947563782[86]):0.11988767082453308244[83]):0.05730440186251870016[46]):0.05888257089393352656[39]):0.05217857362657904452[25],(2667905805:0.45099960238644321020,
(2634852957:0.17865325214310753821,
((2595260104:0.33724230353611761579,2690566818:0.23594851290720328385):0.12314778992151030612[86],2690565850:0.24141642254026726699):0.04528580313322643630[47]):0.25161660722791623357[100]):0.08103975278911711155[38]):0.03187533840774750549[24],
(((2681880531:0.16120985510246160777,GAN75770:0.11019159838403422280):0.06203814410262806223[85],081432845:0.15070171946055418988):0.15476655413558768060[99],(Cabin_B32_k99_1224485:0.12699067613881123706,
(2585097958:0.08401467955788667619,
(2534529065:0.00089598436100228412,2558109243:0.00553109343856977246):0.07081832096626186623[99]):0.17625308921199653311[100]):0.11876054644969205598[96]):0.45040017500877904677[100]):0.04574047625845377441[42],2582914434:0.64165223896099976564):0.04743153355646546887[20]):0.04977413948222760703[7]):0.05092044749769542022[26],
((((2649214640:0.00000100000050002909,OBS09422:0.00259526116094839412):0.05743814969378976953[99],
(2722620737:0.04059305181911025684,2719774481:0.05603092672033881810):0.02981551567914987946[85]):0.36776171045740069232[100],((2649215769:0.00000100000050002909,OBS09238:0.00250742292936124425):0.08633381042941691563[100],
(2722620513:0.04575474556223034506,2719774666:0.11753327753916927023):0.08787193770629014855[98]):0.48349220085483601839[100]):0.26152265488250142633[100],(2684795571:0.17618461569566626346,
((((065414354:0.00000100000050002909,2619155989:0.00205812367993083367):0.00828036651517357822[81],064218474:0.01816782262483278981):0.01458811640483644555[57],
(Atf_SS3:0.05454941021994004491,071182560:0.02997068971593441766):0.00774903988515243843[32]):0.02747671189690064839[61],(CCA63059:0.01015616546693210061,
((Atf_23270:0.00000100000050002909,2510458231:0.00407405520995191613):0.00202962752776074439[96],CAA07031:0.00808305599850438032):0.00413511785695195970[91]):0.02244110131387140605[94]):0.16122894308970509014[100]):0.49381142283696671802[100]):0.08470141377398801574[77]):0.06886536162942552552[29]):0.20843464112520729281[98]):0.22051460335400802082[97],(((((((((OGW13864.1:0.00285167409638152581,2710551439:0.00269452810077779829):1.09443325915573774765[100],
(((2650832612:0.00000100000050002909,264862327:0.00000100000050002909):0.62015963660375972299[100],26505757:0.22957528358592688145):0.23102368035905068711[97],OIO24001.1:0.46168656501316091001):0.64533533254052477535[100]):0.20393768558082522868[45],
(((OGP25644.1:0.44030402429354770222,OGP33174:0.31777225071532666512):0.24385254365645425700[98],
(CUS31318.1:0.39895157353933791144,2691458828:0.23517617050116979005):0.31725181196384127924[99]):0.34380527412675598864[100],OIO90217.1:1.10593475259134765842):0.34841660155049158920[96]):0.10551354904391480050[15],
((((OGV97683.1:0.45220188371517078263,OGQ29950.1:0.55710269337635487386):0.09663508755060845612[54],OGH61190.1:0.60238279052188203888):0.18788613385889402219[91],
(KXK11823.1:0.57177173524883073696,OGB90449.1:0.41947023835342855946):0.17723961808510835247[46]):0.669613161932

87756526[100],OGC96250.1:1.01794940975481007506):0.19462457743057479109[32]):0.03770219731983394384[3],
((025322500:0.50703499109593541672,
((((643639157:0.16881954490892370213,640550771:0.13155081086790992861):0.15009762210134452998[99],
(2651058580:0.33810963661185250428,OGU15740.1:0.45506590874812513636):0.08043909965727513711[54]):0.087456814648
88627188[73],((2722024227:0.13603708212553711210,(642769344:0.11571497979508843512,
((644868508:0.07493853821860338438,
(085813664:0.00000100000050002909,GAW67405:0.00000100000050002909):0.01705863120336845867[100]):0.0338498673124
3382757[69],2524445534:0.04525172154602456753):0.13122478698661682595[100]):0.08109027761095151388[99]):0.070372259
23326137500[93],
(649931631:0.23793589095428585578,2583008628:0.15303347082798374190):0.03920062479833117536[47]):0.17765586769695
390634[99]):0.16257755398442103045[97],(066728662:0.23876986662397961525,
((328735142:0.00335573157528353981,085009218:0.01501443699854966909):0.03541150987050984550[90],3287342504:0.0820
8133739010159413):0.19993666241579313714[100]):0.29029024702936406799[100]):0.11400052605638731629[74]):0.669149402
80763235414[100],(((2650853361:0.31820402484022908274,
(OGQ79863:0.54610523671062938256,OLE63789.1:0.77675366224775066470):0.15649440182913754160[53]):0.57143833621269
035650[99],((((OIO38763:0.21691569277641450930,2710542614:0.28544447576442366632):0.59128483704768441953[100],
((OGX37834.1:0.57869923618377372421,(265008937:0.70199282524223338697,
(2711329036:0.00305450560829520403,OGL49924.1:0.00000100000050002909):0.44557528677132995920[100]):0.13213521407
820530418[85]):0.13846615330193193238[50],((2722154038:0.37465561333755209583,(2713299105:0.43651995932372195108,
(OGU80169.1:0.00000100000050002909,2709276126:0.00000100000050002909):0.28846595380045636503[100]):0.05895289806
623051770[75]):0.24873682175954986429[100],
((2711002114:0.01842578937587047161,OGW08671.1:0.04223684048346521003):0.29936245919168158469[99],
(2710483613:0.14860322543082793434,2712901129:0.07429988225755108922):0.39308909686642679038[100]):0.128818516278
80228084[90]):0.16351186169325421238[65]):0.04712369347403475711[21]):0.09491061400223316236[16],
((OFW08565.1:0.56345407566400140809,(ODS30754.1:0.65899235045042059422,(((OHB93689.1:0.41035506186519471461,
(OHB34676.1:0.09066734507697993273,OHB35667:0.05781781402084524429):0.27529806949376806058[100]):0.140679194279
28016196[98],
(KHE93669.1:0.14700440626752492301,2713121283:0.16208779338452600216):0.32409909585684121280[100]):0.095065292355
56356039[80],2709355360:0.45185714810177396439):0.14268178675509815556[85]):0.08475579162028383517[58]):0.303397548
85472147716[99],(32872071:0.90718451689526680148,
(((((2649221103:0.98368801021155571895,265118736:0.11416769478617383449):0.08875994218829394433[39],2650871304:0.17
014167048046996422):0.13877862847883387643[52],OGU38237:0.28920340898138657826):0.10676920995844135787[71],
(2709619503:0.30241751650359699477,
(OGU76319:0.12253823674720887293,OGU32991:0.26349258104487310872):0.19099427108496030980[99]):0.074238623019250
18836[60]):0.20036280910174356085[90],OFW11146.1:0.46695119850614558166):0.15538940739721698869[86]):0.11431623682
286030930[35]):0.06653063891850295730[20]):0.05682267988346590554[8],((265080367:0.39816928353073804958,
(2709736372:0.00312944210556477097,OHB77915.1:0.00000100000050002909):0.31859113232183466913[100]):0.37925052666
434216908[100],2540319154:0.83284999633185441414):0.06890190444229357669[14]):0.08383166937119201911[21]):0.0700131
1628730938486[26],OGQ89384.1:1.01219097270128610688):0.12423800777515262017[53]):0.17166648656070671031[28]):0.093

96981388694090886[3],
(((OLB32618.1:0.05079494286190432140,OLB40443:0.05175691581110014594):0.93405959171813768283[100],
(((((EES53235.1:0.33313396759480190523,(EES51436.1:0.08992686187936040076,(2611885541:0.15547247024932081549,
(RealCyt572:0.00000100000050002909,
((2655039944:0.00000100000050002909,EDZ39515.1:0.00000100000050002909):0.00000100000050002909[32],U4QTB2_9:0.000
0010000050002909):0.00000100000050002909[82]):0.06526883159469747120[100]):0.08087810520641966483[97]):0.152760755
62226354432[100]):0.67618341728171349470[100],
(((2727864774:0.05404008002046602199,ODT45346.1:0.05285061055330626117):0.10778536282504007360[100],649661898:0.0
2460362872177131177):0.26760368279949131587[100],2727868618:0.30321070619218076780):0.14862993569913840464[98]):0.
11725042284967665540[93],
((OLC18796.1:0.00000100000050002909,OLC72332:0.00000100000050002909):0.67299893822944112376[100],
(((OLB90679.1:0.25313142436208446728,OLD58389:0.30947320944261008657):0.12812585657261138516[94],
(011521241:0.32707471951876099237,OLB27656:0.34673876980339390919):0.07393974242203905922[71]):0.312098296284814
50259[100],2522502658:0.54656405870971436212):0.10915402489200284886[71]):0.16581499577070890616[72]):0.33896894241
650915580[100],OGF68266.1:0.85854706470600783419):0.17211969057682185991[79],((((072151455:0.00000100000050002909,
(072149663:0.00000100000050002909,CUS96789:0.00312154074792892269):0.00311569052596610526[47]):0.000001000000500
02909[4],
(CUU10472:0.00313027455755346927,CUS82647:0.00000100000050002909):0.00312053542676027492[34]):0.103773143468470
95472[100],
(075426666:0.06359064693943518309,CUT00941:0.08413500456174276276):0.03344768762932387451[78]):0.926047193421108
86071[100],((2596636710:0.32439527189559225206,(012676967:0.27097133680177259984,(32874761:0.31803885655619190675,
((328732890:0.17766198267455973747,(OJZ18266:0.10025828479495306478,
(ODU94539:0.11742940948846029847,007080427:0.10195642440208109447):0.09571032067522122766[100]):0.07874905674210
118389[98]):0.06439106094414005776[76],
(((073271468:0.06595819029523138088,SDZ87414:0.04258659457680973731):0.10982097318046793455[100],SDJ55071:0.24175
128829515873941):0.09198309417078084915[99],068616321:0.28533200008653925028):0.05066524090788755014[25]):0.050643
75167703073083[44]):0.06349241281612062748[62]):0.12684395354313537929[76]):0.46475197574404619738[100],
(((KPK59851.1:0.34320021997730837393,KPK07258:0.33111310854065506559):0.31946873128538033626[100],
((014854829:0.26071610857609717193,((073171211:0.34563421683714151689,
(OFY64614:0.18599868557675949821,OFX44164:0.22419806337075537384):0.22281363562082825980[100]):0.072709325352391
07986[69],OGU57542:0.44167767786508693728):0.07661773481772314598[46]):0.11428584647676423569[84],
(((OGD12889.1:0.08109263760419388456,
(OGD16786:0.00426979490865785852,OGD29408:0.00177771384937760188):0.08795364174238223809[100]):0.34531098725957
198869[100],
(OGU31961:0.01245630892101660774,OIP55017:0.00000100000050002909):0.17080443412296320016[100]):0.087581562919640
90678[47],(OQY74200:0.23290860347948072473,
(OGU36629:0.00632595648266069677,OGU83861:0.00000100000050002909):0.23506909260899105929[100]):0.06065783634978
486744[45]):0.07055109406491880120[47]):0.16968587668319751183[87]):0.21861939823585216369[99],048494016:0.602561191
94136736894):0.13861643042185695096[78]):0.33748861557678211387[100]):0.29146420584294896461[78]):0.081746802328472

42323[50]):0.14030845887754414547[27],
(((((312165750:0.07987356500289009842,312116498:0.14378865250196423697):0.05955720613941919289[93],312214575:0.2035
3061094898880490):0.03720197390534175225[51],
((312240224:0.08583383316237097604,312160702:0.03872927753950803492):0.16267216817542598517[100],
((639880235:0.07299251529374310710,
(GAV20625.1:0.08962357774908498442,3122231637:0.11165882863192329311):0.03874698808253631416[70]):0.0263672660768
2705892[64],
((3122031242:0.00260392684815838544,S19_NODE_894_16:0.00000100000050002909):0.11913330880550512814[100],
(3121461290:0.07489819890681849446,(312159909:0.05335335111957046489,
(((3121561583:0.04298403739415288666,3121591956:0.01926171329395243853):0.00984921936648597805[22],
(312128345:0.02450160183326387936,312106304:0.00551388083753183867):0.03121289176782974031[98]):0.015160953147930
13300[32],(312160597:0.02774243166986783971,
((312250265:0.00280513154752221744,312234557:0.00259029698943342426):0.02748663904125380961[96],
(312198583:0.00502853147651988620,312235820:0.01191738596556302966):0.02617038966427182034[92]):0.013060400056443
30294[65]):0.01044711981381055317[66]):0.01929083441028402873[39]):0.02291773361541881229[29]):0.0209711553512413571
8[36]):0.10742672895332318339[95]):0.03322917688089106064[41]):0.02788546620540474988[43]):0.03073508886795885131[65]
,(3122011704:0.13924999908629806522,((312134101:0.00377085174910061832,(2583143739:0.00508296491568720459,
((31214176:0.03431939797646707535,2617305523:0.01165175363096835996):0.00269153170724720529[56],312231926:0.01470
760579573725593):0.01726803255581721777[88]):0.02003933113412333447[70]):0.14313326488957830995[98],2525812737:0.15
831440617084757694):0.09739941127530717113[94]):0.03153430778350976155[55]):0.84038243694463532485[100],265021150:
1.25078020396041922346):0.09322474186168974719[18]):0.10176205421774656146[2]):0.10133760375212944038[1],
((((2727863661:0.28742623624255009274,
(((041186371:0.00000100000050002909,649658983:0.00000100000050002909):0.02153741939360761734[100],
(SLM43750:0.00000100000050002909,080878845:0.00000100000050002909):0.02346843125874329555[100]):0.05608328385612
946360[100],2727866385:0.08060599393085696174):0.13817384661242299027[97]):0.43691833812941405224[100],
((2651081824:0.31272349190724096912,
(Tenderia:0.16241603832256490403,3287751955:0.15549622137095886476):0.13413402756588299614[98]):0.1747561624006885
0282[94],32881481:0.31102341753889550802):0.25998076225996896715[97]):0.69404784246938810988[100],
(((OGQ64145:0.00000100000050002909,2712741716:0.00267476158732185988):0.25370332791520044946[100],
((OGS90975:0.00583968149928137080,OGT17989:0.00250515921484897094):0.06612364803333158070[100],OGT15083:0.06240
715541720747578):0.24476734978460618075[100]):0.64574408222847101690[100],(313189204:1.53001958441155538893,
((OGM98442.1:0.63920172960634491499,
((OIO16315:0.35683843983465024507,OGU30417:0.18515541726567935621):0.03118028002713716870[49],
((OGU32428.1:0.29353606103911961123,OGU13705:0.24204952262507700711):0.03984972937156015110[51],OGU69631:0.1842
6856269660901733):0.05322316225672393125[72]):0.22891438043472078778[99]):0.12372274660697327175[90],
(((OGQ05831.1:0.12174457420009041075,OGQ44746:0.22701507750243879458):0.31319553137396299869[100],
(OIO04201:0.57852843680365428902,
(OGR57352:0.17594834909984752302,OGR83528.1:0.15692554170957453974):0.31292857665244661058[100]):0.118700674358
40077341[80]):0.32013097511020111030[100],048493423:0.47503890594578784823):0.08398734320931480757[55]):0.515126924

25242001537[100]):0.18041616087734638207[22]):0.18675826654380250913[6]):0.09209545117828360494[3],
((OFV98020.1:0.92635137907178444205,
(OGW55307.1:0.00938457451202261324,2721826394:0.01269885513699576043):0.48386757837173799413[100]):0.51975959281567618575[98],(((2712994656:0.35526908711401933294,OGR96280.1:0.37542129850531574808):0.55970086457784751222[100],
(3288521941:0.99091828942313919804,
((((265055679:0.33884500884164514556,270410515:0.43230379192955792345):0.18440476435447003545[99],
(OGI37867:0.48130610197384027238,328775635:0.29980925582518419548):0.15540980352521649444[99]):0.09710236972620428508[79],((2559016914:0.38750654132825773646,(265055293:0.22328963725551020936,
(264768335:0.00000100000050002909,2648361733:0.00000100000050002909):0.18773892110861983840[100]):0.35201038983884169742[100]):0.13408611947493548344[59],
(3288641505:0.18592570580932810120,270425764:0.21426434613573566712):0.41127743003755645734[100]):0.08810553557347651499[58]):0.08861335763972376378[44],(2709517489:0.65047730199981379506,
(Cabin_A27_k99_127935:1.04137418978752860355,3287702490:0.24335187163841520697):0.34071164473232812275[96]):0.06149620602859773300[46]):0.10562555347700310071[64]):0.19423324692344900999[93]):0.18510687260593031156[88],
(OFW51332.1:0.76075663469990295606,
((OGV99236:0.21075002680353374651,OGW04464.1:0.18999662207912995648):0.45902750141805942485[100],
(OGV99616:0.25269014659235761178,
(2711002013:0.09151014924270796380,OGW05040:0.10098209762949852952):0.13123736473509564626[99]):0.44468376695972450241[100]):0.39467190883888131347[100]):0.51496158226986687989[86]):0.04130886696659746837[20]):0.15371481794900263007[27]):0.05231753699126667073[0]):0.05394987306327060639[8],
(KRT68508.1:0.93300172799430869386,2651051644:0.81597867107691057775):0.35898511815473421738[49]):0.07193637254655843583[29],2662521112:1.12684987768749422798):0.08321251411955195298[61]):0.08850102868817018298[45],
(077280302:0.78295831519138781029,2682287270:1.26581030034579056220):0.32370605271986901963[88]):0.27889304431836531295[97],
(((Cabin_C38_k99_99254:0.22684676673614542763,Cabin_C38_k99_46640:0.37419905110198214970):1.05969848920779852186[100],Sulfuro_AR:0.29283324481322564603):0.59353703384820000188[97],(OIO16638.1:0.39721740990380211000,
((2710783000:0.00000100000050002909,2712826751:0.00000100000050002909):0.18434636410217689062[100],
(SulfPC0866:0.38173716263948920258,OIP54822.1:0.26763155719227726959):0.08682719773798634744[48]):0.06827680276063166631[44]):0.31930537505908296314[100]):0.21527155400437367194[96]):0.22983732352871999449[100]):0.16539926226519671704[99]):0.07717134387017134112[59]):0.11298129185194526192[57],(OIO78393.1:0.36062027105083044320,
(((2709849208:0.25242825522932255122,
(endoRiftia:0.00000100000050002909,endoTevnia:0.00000100000050002909):0.32957587617175732042[100]):0.06618053297730802642[43],
((((2713759214:0.17092297965903979762,OGG97149.1:0.43218795534878323705):0.22241759731225099750[99],270416507:0.62648064810166725902):0.15400374261382815400[97],(2603819388:0.17060963272068654928,
(068987405:0.01489167330906817667,
(069002917:0.00000100000050002909,069015424:0.00245025512428013320):0.00000100000050002909[78]):0.03972966535179347364[74]):0.35757651601153866983[100]):0.15096701191969266742[98],
(2710309193:0.22437698663145785871,OGI47527.1:0.18606094678202952930):0.19217260263450156033[100]):0.064841486924

27295357[48]):0.07957944396273587684[69],
((((((2582857759:0.12706104048074465251,2582907738:0.17009014593454155340):0.16130809006624427515[100],
((Cluteo_273:0.04323880704037203621,KZK73968.1:0.04574607654689389485):0.23077988674976132599[100],
(Cfer_13031:0.03849366993156679018,076792910:0.03123775376625834502):0.18315263441035950498[100]):0.0581939643181
8512488[66]):0.27573801174780399048[98],2709297025:0.22393336594949445062):0.04847833489790939754[25],2701360880:0.
34907786470754659236):0.04596853976767736188[15],((2650971371:0.36425237919400016429,
((264931153:0.02658807099683996311,265136474:0.07478149532028577351):0.05891443320497109781[99],
((264872627:0.02281938143312504247,2648211797:0.07507115388747535867):0.03737059672232376922[100],
((265112973:0.02405119103457615398,
(2650971901:0.00000100000050002909,264813593:0.00000100000050002909):0.03482574008160208706[100]):0.0553219094055
8447123[99],
(266254112:0.00000100000050002909,2650911651:0.00000100000050002909):0.04705516063801622878[100]):0.0281560987749
6906208[87]):0.04858770836357311634[97]):0.20668548633714123319[100]):0.06878599194287166041[58],
(2712900913:0.13206523568400591051,2710482821:0.12472628514279386625):0.11412812035906241825[97]):0.0551989662137
5754847[43]):0.05500906869811698213[26],
(2709740544:0.27447938988541692451,2709877489:0.27382677324339865788):0.05097086665501222857[49]):0.0813352048040
4776709[38]):0.05178841964618108307[20]):0.08127223311249014148[35]):0.08907568382152730724[63],
(((2716601524:0.23594717273295084947,(646687914:0.31860297080060206998,
(2711279851:0.12619652281653301329,2711309673:0.16641954887951113107):0.12567613067898225632[99]):0.0772922855403
0230514[95]):0.06649539386210250036[92],
((2709509328:0.13561766334285713098,2711310829:0.28106632733964392878):0.05573533411063347903[86],
(NDFO_KS:0.29866545600075938838,
(2709235129:0.04372753796073992894,2709232020:0.04377082142160122064):0.11484120563749766752[99]):0.0607540802337
4168076[47]):0.02624827214251891291[26]):0.03639144529018051993[33],(2700974797:0.31857747470047348415,
(2695407354:0.14639293930606897542,
(2716621633:0.05019410264518296694,2724742866:0.00132948518534349602):0.20200310887368130852[100]):0.116366268326
94940692[100]):0.14513066688825654316[100]):0.13801776769235030162[100]):0.06026635390769390088[60]):0.0768643077736
1090427[86]):0.12238341964666518691[99]):0.06459312104900641338[85]):0.09743727674654956150[98],KXS32102.1:0.2914033
6525520565969);

File S4. Metagenome assembly, mapping, and binning statistics.

Analysis	Quality Control			Assembly						Mapping			
**Assembly**	**Number of Raw Reads**	**Both Surviving**	**Both Surviving (%)**	**Contigs**	**Total Length**	**Minimum Length (bp)**	**Maximum Length (bp)**	**Average Length (bp)**	**Median Length (bp)**	**Reads Mapping Input**	**Reads Mapped (%)**	**Bases Binned**	**Bins**
**Combined**	n/a	n/a	n/a	74903	639525001	1000	140779	2326	2531	204852293	54.32%	265006380	120
**316A**	81646666	63854031	78%	50449	147593125	1000	140108	2926	3853	63854031	56.02%	72679942	38
**316B**	101886314	82746190	81%	136051	273880342	1000	202162	2013	1979	82746190	55.76%	89275406	32
**316C**	74021852	58252072	79%	118786	305336439	1000	140779	2570	3060	58252072	53.91%	150139920	66

File S5. Schematic of the METABOLIC and DRAM outputs summarizing the major metabolic pathways in each MAG.

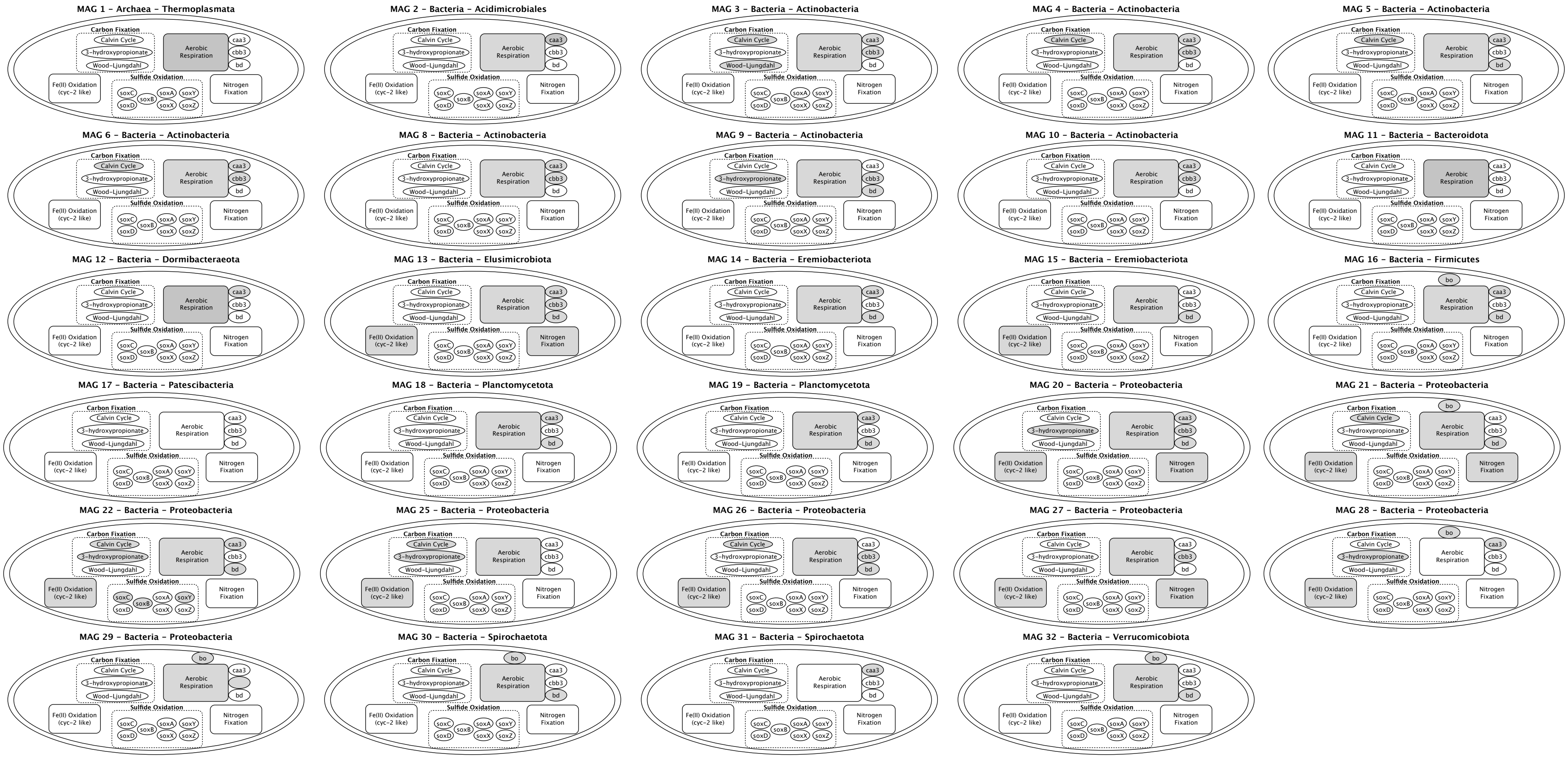

File S6. Supplemental dataset: Output from DRAM showing presence or absence of gene necessary for metabolic pathways for each MAG.

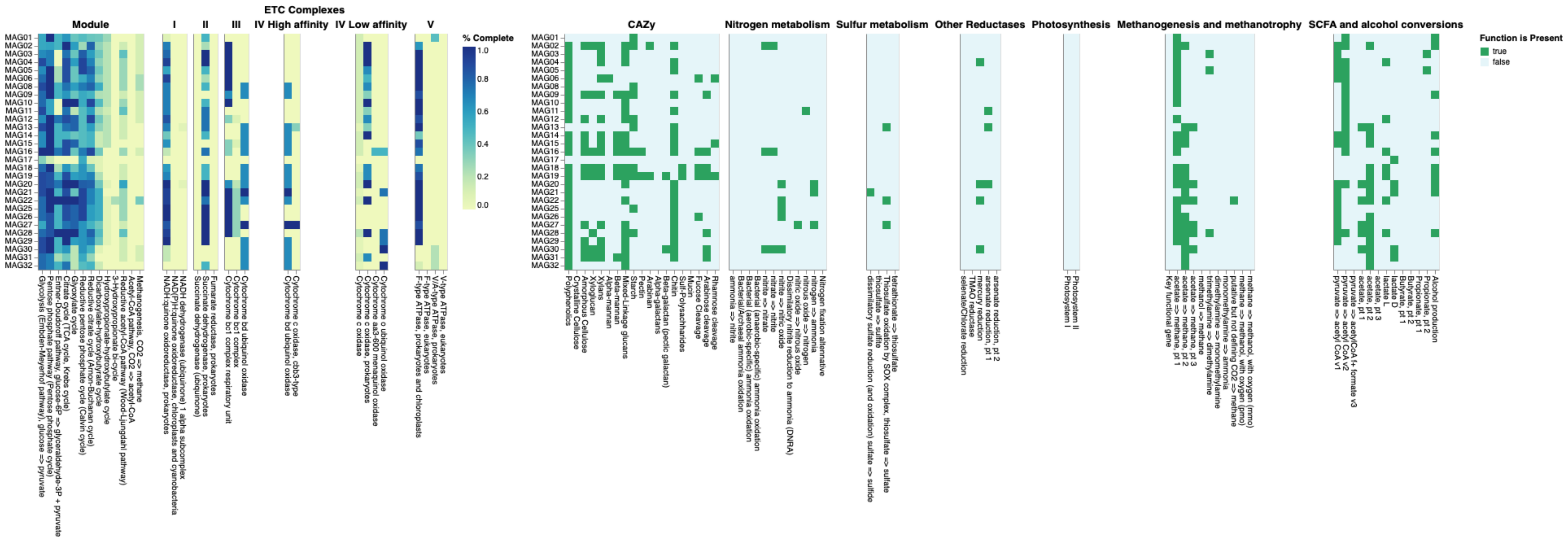

Supplement: Supplemental file 1 — Files S1 to S6. Download AEM.00772-21-s0001.pdf, PDF file, 0.6 MB [file aem.00772-21-s0001.pdf]
